# Synthesis, preliminarily biological evaluation and molecular docking study of new Olaparib analogues as multifunctional PARP-1 and cholinesterase inhibitors

**DOI:** 10.1080/14756366.2018.1530224

**Published:** 2018-11-14

**Authors:** Cheng-Zhi Gao, Wei Dong, Zhi-Wen Cui, Qiong Yuan, Xia-Min Hu, Qing-Ming Wu, Xianlin Han, Yao Xu, Zhen-Li Min

**Affiliations:** a Hubei Province Key Laboratory of Occupational Hazard Identification and Control, Wuhan University of Science and Technology, Wuhan, China;; b College of Pharmacy, Shanghai University of Medicine & Health Sciences, Shanghai, China;; c Barshop Institute for Longevity and Aging Studies, University of Texas Health Science Center at San Antonio, San Antonio, TX, USA;; d College of Life Science and Health, Wuhan University of Science and Technology, Wuhan, China

**Keywords:** PARP-1 inhibitor, Olaparib, AChE, BChE, Alzheimer's Disease

## Abstract

A series of new Olaparib derivatives was designed and synthesized, and their inhibitory activities against poly (ADP-ribose) polymerases-1 (PARP-1) enzyme and cancer cell line MDA-MB-436 *in vitro* were evaluated. The results showed that compound **5l** exhibited the most potent inhibitory effects on PARP-1 enzyme (16.10 ± 1.25 nM) and MDA-MB-436 cancer cell (11.62 ± 2.15 μM), which was close to that of Olaparib. As a PARP-1 inhibitor had been reported to be viable to neuroprotection, in order to search for new multitarget-directed ligands (MTDLs) for the treatment of Alzheimer’s disease (AD), the inhibitory activities of the synthesized compounds against the enzymes AChE (from electric eel) and BChE (from equine serum) were also tested. Compound **5l** displayed moderate BChE inhibitory activity (9.16 ± 0.91 μM) which was stronger than neostigmine (12.01 ± 0.45 μM) and exhibited selectivity for BChE over AChE to some degree. Molecular docking studies indicated that **5l** could bind simultaneously to the catalytic active of PARP-1, but it could not interact well with huBChE. For pursuit of PARP-1 and BChE dual-targeted inhibitors against AD, small and flexible non-polar groups introduced to the compound seemed to be conducive to improving its inhibitory potency on huBChE, while keeping phthalazine-1-one moiety unchanged which was mainly responsible for PARP-1 inhibitory activity. Our research gave a clue to search for new agents based on AChE and PARP-1 dual-inhibited activities to treat Alzheimer’s disease.

## Introduction

Poly (ADP-ribose) polymerases-1 (PARP-1) is a pivotal nuclear enzyme ubiquitously expressed in eukaryotic cells. It can detect DNA single-strand break and catalyse the addition of ADP-ribose units to acceptor proteins, thus facilitating DNA repair process and promoting cell survival^1^^,^^2^. Inhibition of PARP-1 accelerates the damage of injured DNA, which especially leads to synthetic lethality in DNA-repairing-deficient cancer cells, for example BRCA1/2-deficient cells. Therefore, PARP-1 has become an attractive antitumor target^3–5^. PARP-1 inhibitors can be utilized not only as a single agent for the treatment of BRCA1- or BRCA2-deficient cancers, but in combination with DNA-damaging therapeutics (radiation or chemotherapy) to improve their potencies by blocking DNA-repairing process. Then, PARP-1 inhibitors have been extensively investigated as potential anticancer drugs^6^^,^^7^.

In addition to being used for cancer treatment, recent researches indicate that PARP-1 inhibitors have potentially therapeutic value in Alzheimer's disease (AD)^8–10^. PARP-1 utilizes nicotinamide adenine dinucleotide (NAD^+^) as substrate to catalyse the transfer of ADP-ribose units to nuclear target proteins upon oxidative stress and DNA injury. Over-activation of PARP-1 will lead to cellular depletion of NAD^+^ and ATP, and then, energy deficiency slows glycolysis and mitochondrial respiration rates, resulting in controlled nerve cell death. This PARP-related specific neurodegenerative mechanism was termed as parthanatos^10^. Meanwhile, over-activated PARP-1 will increase its interaction with transcription regulators such as nuclear receptors, sirtuins, and other metabolic transcription factors. Further, it will also lead to the impairment of mitochondrial function that seems to be an early and critically important event in the pathogenesis of AD^9^^,^^11^. Although the role of PARP-1 in human AD is not fully elucidated and many mechanisms are under investigations, accumulating evidences suggest that PARP-1 inhibitors are viable to neuroprotection and PARP-1 has emerged as a potential therapeutic target for AD^12^^,^^13^. As for AD, it still imposes a great threat to human health. Currently, most of the approved drugs for AD treatment are acetylcholinesterase (AChE) inhibitors, namely donepezil, tacrine, rivastigmine and galantamine. Nevertheless, these drugs modestly alleviate the symptoms but cannot cure brain damage or stop neuronal degeneration^14^. Therefore, discovery for effective anti-AD drugs remains an enormous challenge to medicinal chemistry communities. In fact, in addition to AChE, there is another cholinesterase, butyrylcholinesterase (BChE), in the synaptic gap of neuronal cells to efficiently hydrolyse acetylthiocholine (ACh), and its inhibitors have been reported to be more beneficial to AD in later stages of AD than AChE inhibitors^15–17^. In the light of the multifactorially pathological mechanisms of AD, developing multitarget-directed ligands (MTDLs) may be a more promising approach for its treatment. Some researches and reviews of MTDLs for AD have already been reported^18^^,^^19^, but almost none of which is related to both PARP-1 and cholinesterase inhibitors^20^. In this regard, the PARP-1 and cholinesterase dual-targeted inhibitor of AD are worthy to be investigated.

**Figure 1.**
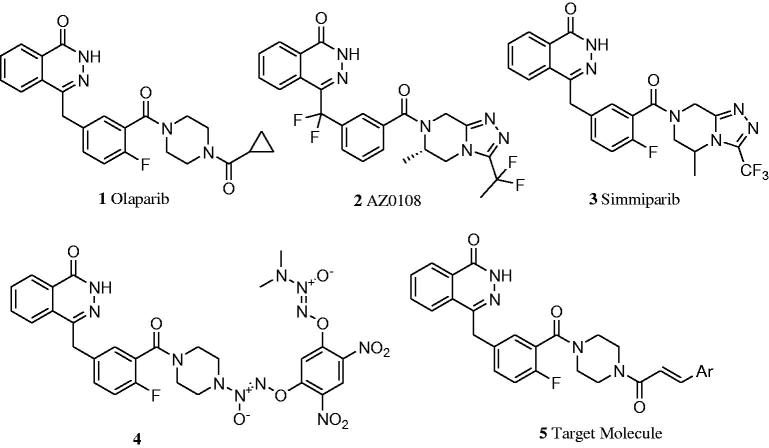
The structures of Olaparib analogs and target molecules.

Olaparib is the first-in-class PARP-1 inhibitor marketed in 2014 as monotherapy for advanced BRCA-deficient ovarian cancer. However, its anticancer activity was not potent enough^7^^,^^21^, and it showed dose-limiting toxicity that was more pronounced than that seen with the chemotherapeutic agents alone when it was utilized in combination with chemotherapeutic agents for cancer treatment^22^. To overcome these shortcomings, several modifications based on structural skeleton of Olaparib have been reported (Figure 1)^21–24^. The reported structure–activity relationship (SAR) revealed that 4-benzyl phthalazinone was the core scaffold responsible for moderate PARP-1-inhibiting potency, and a substituted piperazine at the meta position of benzyl moiety was conducive to enhancing activity and maintaining good oral bioavailability^7^^,^^25^. In view of the important therapeutic value of PARP-1 inhibitor for cancer and potentially for AD treatment, it was interesting and deserved to further explore structural optimization of Olaparib based on the existed SAR. With this aim in mind, we decided to keep the 4-benzyl phthalazinone group unchanged and replace the cyclopropane group of Olaparib by substituted aryl vinyl ones to constitute 3-aromatic α, β-unsaturated carbonyl moiety. On the one hand, the aryl vinyl groups are hydrophobic, just like cyclopropane group, which is helpful to improve oral bioavailability^7^; on the other hand, 3-aromatic α, β-unsaturated carbonyl moiety is widely included into natural products, especially within chalcone framework that possesses a variety of bioactivities, including anticancer, inhibition of cholinesterases and neuroprotection^26–28^. Through the structural optimizations, we hoped to find a more potent PARP-1 inhibitor, at the same time, in order to explore new MTDLs for the treatment of AD, the inhibitory activities against AChE and BChE enzymes of these compounds were also evaluated. Here, we described the synthesis and bioactivities assays of 15 Olaparib derivatives; meanwhile, molecular dockings were conducted to investigate their interaction fashion with the corresponding proteins.

## Materials and methods

### Chemistry

All reagents and solvents were obtained from commercially available sources and were used without further purification. Reaction progress was monitored using analytical thin layer chromatography (TLC) on precoated silica gel GF254 (Qingdao Haiyang Chemical Plant, Qing-Dao, China) plates and spots were detected under UV light (254 nm). Melting points were determined on a WRS-2B digital melting point apparatus and uncorrected. IR spectra were recorded using a Nicolet 380 Fourier-transform infrared (FTIR) spectrophotometer (Thermo, USA) from KBr pellets. ^1^H NMR and ^13^C NMR spectra were recorded on a Bruker AVANCE III-600 NMR spectrometer (Bruker Biospin Co., Switzerland) with tetramethylsilane (TMS) as the internal standard and CDCl_3_ or DMSO-d_6_ as solvent and known chemical shifts of residual proton signals of deuterated solvents (^1^H NMR: *δ*: 2.50 for DMSO-d_6_ and *δ*: 3.33 for H_2_O) or carbon signals of deuterated solvents (^13^C NMR: *δ*: 39.52 for DMSO-d_6_) as internal standard. MS (ESI) measurement was conducted on an Agilent 1100 LC-MS spectrometer (Agilent, Palo Alto, USA).

### Dimethyl (3-oxo-1, 3-dihydro-isobenzofuran-1-yl)-phosphonate (7)

Compounds **7**, **8** and **9** were prepared according to the literature procedure^24^^,^^29^. A mixture of 2-formylbenzoic acid **6** (2.0 g, 15.0 mmol) and dimethyl phosphate (4.0 g, 36.0 mmol) was heated to 100 °C in oil bath and stirred for 8 h. Subsequently, the mixture was cooled to room temperature and then poured into ice-cold water (20 mL), which was followed by extraction with dichloromethane (3 × 30 mL). The combined organic extracts were dried over MgSO4 and were evaporated in vacuum to produce a white solid which was crystallized in ethanol to yield 2.25 g compound **7**. Yield: 62.3%, mp: 96.1–98.2 °C. ^1^H-NMR (CDCl_3_, 600 MHz), *δ*: 7.93 (d, *J* = 7.68 Hz, 1H, ArH), 7.76–7.70 (m, 2H, ArH), 7.58 (t, *J* = 7.56 Hz, 1H, ArH), 5.70 (d, *J* = 10.98 Hz, 1H, CH), 3.91 (d, *J* = 10.92 Hz, 3H, OCH_3_), 3.58 (d, *J* = 10.62 Hz, 3H, OCH_3_).

### 2-Fluoro-5-[(3-oxo-2-benzofuran-1-ylidene) methyl] benzonitrile (8)

To a mixture of **7** (4.3 g, 17.8 mmol) and 2-fluoro-5-formylbenzonitrile (2.6 g, 17.5 mmol) in anhydrous THF (40 mL) was added triethylamine (1.8 mL, 13 mmol) dropwise in 30 min, and the temperature was maintained below 15 °C. The reaction mixture was slowly warmed to room temperature. The progress of the reaction was monitored by TLC. After completion of the reactions, the reaction mixture was then concentrated *in vacuo*. The residue was slurried in water (50 mL) for 30 min, and the solid was collected by filtration, was washed with diethyl ether (2 × 10 mL) and was dried to afford 3.6 g of **8** in 78.5% yield. The material was carried forward without further purification.

### 2-Fluoro-5-[(4-oxo-3H-phthalazin-1-yl) methyl] benzoic Acid (9)

To a stirred suspension of **8** (2.1 g, 8.0 mmol) in water (20 mL) was added aqueous NaOH (10 mol/l, 3.5 mL) with subsequent heating to 90 °C for 1 h. The reaction mixture was cooled to 70 °C into which hydrazine hydrate (5.4 mL, 94.0 mmol) was added, and the mixture was stirred for 18 h at 70 °C before TLC indicated the reaction was completed. The mixture was cooled to ambient temperature and was acidified with HCl (4 mol/L) to pH 4, and then, the suspension was filtered and was washed with diethyl ether and dried to get 2.2 g of **9** as a light red powder. Yield: 93.7%, mp: 208.4–210.1 °C。^1^H-NMR (600 MHz, DMSO-d_6_), *δ*: 13.05 (s, 1H, COOH), 12.56 (s, 1H, NH), 8.26–8.20 (m, 1H, ArH), 7.94 (d, *J* = 8.0 Hz, 1H, ArH), 7.91–7.84 (m, 1H, ArH), 7.83–7.75 (m, 2H, ArH), 7.58–7.50 (m, 1H, ArH), 7.20 (m, 1H, ArH), 4.32 (s, 2H, CH_2_).

### General procedure for preparation of intermediates 13a–o

Compounds **10a–o**, **11a–o**, **12a–o** and **13a–o** were prepared according to the literature procedures^29^^,^^30^. To a stirred solution of various substituted benzaldehydes (or 2-furaldehyde) (24.0 mmol) in pyridine (15 mL), malonic acid (4.9 g, 48.0 mmol) and ammonium acetate (0.2 g, 23 mmol) were added. The mixture was stirred for 5 h at 85 °C and then cooled to room temperature, slowly diluted with ice water (100 mL). The solution was acidified with HCl to pH 2, and the precipitate formed. After filtered, the solid was washed with water until it had no pyridine smell. It was dried, which was followed by recrystallization from ethanol to give **10a–o** in 50–80% yields. Compounds **10a–o** (8.0 mmol) were dissolved in DMF (0.25 mL) to which oxalyl chloride was added dropwise at 0–5 °C. The mixture was stirred for 1.5 h at room temperature and then evaporated under reduced pressure. The residue **11a–o** was added dichloromethane (DCM, 15 mL) to dissolve and sealed for use in next step. *Tert*-butoxycarbonylpiperazine (1.5 g, 8.0 mmol) and triethylamine (1.1 mL, 8.0 mmol) were dissolved in DCM (15 mL). After dropwise addition of **11a–n** at 0–5 °C, the mixture was stirred for 12 h at room temperature. The mixture was washed with saturated NaHCO_3_ solution (2 × 15 mL), brine (30 mL), dried over Na_2_SO_4_ and evaporated under reduced pressure, and then, the residue was purified by silica gel column chromatography to obtain **12a–o** in 40–70% yields. Finally, **12a–o** (12.0 mmol) was dissolved in DCM (30 mL) to which trifluoroacetic acid (78 mmol) was added, and then, the mixture was stirred for 8 h at room temperature. Water (30 mL) was added to the mixture, which was stirred for another 0.5 h and then separated. The water layer was extracted with DCM (3 × 10 mL), the combined organic layers were dried over Na_2_SO_4_ and concentrated and purified by silica gel column chromatography to give **13a–o** as yellow oils in 50–80% yields^1^. HNMR data of **12a–o** were shown below:

### *Tert-*butyl 4-((*E*)-3-phenylacryloyl)piperazine-1-carboxylate (12a)

A white solid, yield: 63.3%, mp: 131.1–132.2 °C, ^1^H NMR (600 MHz, CDCl_3_) *δ*: 7.68 (d, *J* = 15.4 Hz, 1H, =CH), 7.51 (dd, *J* = 7.8, 1.4 Hz, 2H, ArH), 7.35 (dd, *J* = 8.3, 6.6 Hz, 2H, ArH), 6.84 (d, *J* = 15.4 Hz, 1H, =CH), 3.66–3.47 (m, 8H, NCH_2_), 1.47 (s, 9H, C (CH_3_)_3_).

### *Tert-*butyl 4-[(*E*)-3-(3,4-dimethoxyphenyl)acryloyl]piperazine-1-carboxylate (12b)

A white solid, yield: 55.3%, mp: 124.9–125.1 °C, ^1^H NMR (600 MHz, CDCl_3_) *δ*: 7.87 (d, *J* = 15.6 Hz, 1H, =CH), 7.02 (s, 1H), 6.91 (d, *J* = 15.6 Hz, 1H, =CH), 6.83 (dt, *J* = 17.9, 5.9 Hz, 2H, ArH), 3.80 (s, 3H, OCH_3_), 3.76 (s, 3H, OCH_3_), 3.65–3.62 (m, 4H, NCH_2_), 3.50–3.36 (m, 4H, NCH_2_), 1.45 (s, 9H, C (CH_3_)_3_).

### *Tert-*butyl 4-[(*E*)-3-(4-methoxyphenyl)acryloyl]piperazine-1-carboxylate (12c)

A yellow solid, yield: 56.3%, mp: 127.1–128.8 °C, ^1^H NMR (600 MHz, CDCl_3_) *δ*: 7.90 (d, *J* = 15.6 Hz, 1H, =CH), 7.47 (d, *J* = 7.6 Hz, 2H, ArH), 7.33–7.28 (m, 2H, ArH), 6.94 (d, *J* = 15.6 Hz, 1H, =CH), 3.88 (s, 3H, OCH_3_), 3.68–3.62 (m, 4H, NCH_2_), 3.53–3.16 (m, 4H, NCH_2_), 1.46 (s, 9H, C (CH_3_)_3_).

### *Tert*-butyl 4-[(*E*)-3-(4-methylphenyl)acryloyl]piperazine-1-carboxylate (12d)

A light yellow solid, yield: 58.3%, mp: 149.6–151.3 °C, ^1^H NMR (600 MHz, DMSO-d_6_) *δ*: 7.88 (d, *J* = 15.6 Hz, 1H, =CH), 7.82–7.79 (m, 2H, ArH), 7.59–7.56 (m, 2H, ArH), 6.91 (d, *J* = 15.6 Hz, 1H, =CH), 3.77–3.48 (m, 6H, NCH_2_), 3.21–3.19 (m, 2H, NCH_2_), 2.31 (s, 3H, CH_3_), 1.45 (s, 9H, C (CH_3_)_3_);

### *Tert*-butyl 4-[(*E*)-3-(2-methoxyphenyl)acryloyl]piperazine-1-carboxylate (12e)

A white solid, yield: 58.1%, mp: 114.7–115.8 °C, ^1^H NMR (600 MHz, CDCl_3_) *δ*: 7.91 (d, *J* = 15.6 Hz, 1H, =CH), 7.47 (d, *J* = 7.6 Hz, 1H, ArH), 7.33–7.28 (m, 1H, ArH), 6.98–6.88 (m, 3H, ArH and = CH), 3.87 (s, 3H, OCH_3_), 3.68–3.62 (m, 4H, NCH_2_), 3.53–3.16 (m, 4H, NCH_2_), 1.46 (s, 9H, C (CH_3_)_3_);

### *Tert*-butyl 4-[(*E*)-3-(2,5-dimethoxyphenyl)acryloyl]piperazine-1-carboxylate (12f)

A light yellow solid, yield: 60.2%, mp: 147.0–147.6 °C, ^1^H NMR (600 MHz, CDCl_3_) *δ*: 7.85 (d, *J* = 15.6 Hz, 1H, =CH), 6.99 (d, *J* = 2.9 Hz, 1H, ArH), 6.92 (d, *J* = 15.6 Hz, 1H, =CH), 6.83 (dt, *J* = 17.9, 5.9 Hz, 2H, ArH), 3.80 (s, 3H, OCH_3_), 3.76 (s, 3H, OCH_3_), 3.65–3.61 (m, 4H, NCH_2_), 3.50–3.36 (m, 4H, NCH_2_), 1.45 (s, 9H, C (CH_3_)_3_).

### *Tert*-butyl 4-[(*E*)-3-(4-fluorophenyl)acryloyl]piperazine-1-carboxylate (12g)

A white solid, yield: 62.1%, mp: 143.6–144.1 °C, ^1^H NMR (600 MHz, CDCl_3_) *δ*: 7.64 (d, *J* = 15.4 Hz, 1H, =CH), 7.54–7.45 (m, 2H, ArH), 7.09–7.00 (m, 2H, ArH), 6.76 (d, *J* = 15.4 Hz, 1H, =CH), 3.65–3.32(m, 8H, NCH_2_), 1.46 (s, 9H, C (CH_3_)_3_).

### *Tert*-butyl 4-[(*E*)-3–(4-trifluoromethylphenyl)acryloyl]piperazine-1-carboxylate (12h)

A white solid, yield: 56.2%, mp: 173.1–173.6 °C, ^1^H NMR (600 MHz, CDCl_3_) *δ*: 7.68 (d, *J* = 15.4 Hz, 1H, =CH), 7.64–7.58 (m, 4H, ArH), 6.92 (d, *J* = 15.5 Hz, 1H, =CH), 3.66–3.48 (m, 8H, NCH_2_), 1.47 (s, 9H, C (CH_3_)_3_).

### *Tert*-butyl 4-[(*E*)-3-(3-fluorophenyl)acryloyl]piperazine-1-carboxylate (12i)

A white solid, yield: 55.3%, mp: 121.8–122.2 °C, ^1^H NMR (600 MHz, CDCl_3_) *δ*: 7.62 (d, *J* = 15.4 Hz, 1H, =CH), 7.32 (d, *J* = 5.8 Hz, 1H, ArH), 7.26–7.20 (m, 1H, ArH), 7.03 (t, *J* = 8.5 Hz, 1H, ArH), 6.83 (d, *J* = 15.4 Hz, 1H, =CH), 3.65–3.47 (m, 8H, NCH_2_), 1.46 (s, 9H, C (CH_3_)_3_).

### *Tert*-butyl 4-[(*E*)-3-(4-chlorophenyl)acryloyl]piperazine-1-carboxylate (12j)

A white solid, yield: 58.2%, mp: 187.1–187.5 °C, ^1^H NMR (600 MHz, DMSO-d_6_) *δ*: 7.73 (d, *J* = 8.4 Hz, 2H, ArH), 7.49–7.44 (m, 3H, ArH and = CH), 7.25 (d, *J* = 15.4 Hz, 1H), 3.58–3.31 (m, 8H, NCH_2_), 1.38 (s, 9H, C (CH_3_)_3_).

### *Tert*-butyl 4-[(*E*)-3-(4-bromophenyl)acryloyl]piperazine-1-carboxylate (12k)

A white solid, yield: 65.3%, mp: 207.7–208.4 °C, ^1^H NMR (600 MHz, DMSO-d_6_) *δ*: 7.65 (d, *J* = 8.4 Hz, 2H, ArH), 7.57 (d, *J* = 8.4 Hz, 2H, ArH), 7.43 (d, *J* = 15.4 Hz, 1H, =CH), 7.26 (d, *J* = 15.4 Hz, 1H, =CH), 3.58–3.39 (m, 4H, NCH_2_), 3.32 (s, 4H, NCH_2_), 1.38 (s, 9H, C (CH_3_)_3_).

### *Tert*-butyl 4-[(*E*)-3-(3-nitrophenyl)acryloyl]piperazine-1-carboxylate (12l)

A white solid, yield: 52.4%, mp: 145.1–145.8 °C, ^1^H NMR (600 MHz, DMSO-d_6_) *δ*: 7.80 (d, *J* = 6.6 Hz, 1H, ArH), 7.72 (d, *J* = 15.4 Hz, 1H, =CH), 7.67–7.62 (m, *2*H, ArH), 7.59 (t, *J* = 7.2 Hz, 1H, ArH), 7.45 (d, *J* = 15.4 Hz, 1H, =CH), 3.60–3.39 (m, 4H, NCH_2_), 3.35–3.31 (m, 4H, NCH_2_), 1.39 (s, 9H, C (CH_3_)_3_).

### *Tert*-butyl 4-[(*E*)-3-(4-nitrophenyl)acryloyl]piperazine-1-carboxylate (12m)

A white solid, yield:55.6%, mp: 212.1–213.3 °C, ^1^H NMR (600 MHz, DMSO-d_6_) *δ*: 7.65 (d, *J* = 8.4 Hz, 2H, ArH), 7.57 (d, *J* = 8.4 Hz, 2H, ArH), 7.43 (d, *J* = 15.4 Hz, 1H, =CH), 7.26 (d, *J* = 15.4 Hz, 1H, =CH), 3.58–3.31 (m, 4H, NCH_2_), 3.32 (s, 4H, NCH_2_), 1.38 (s, 9H, C (CH_3_)_3_).

### *Tert*-butyl 4-[(*E*)-3-(3,4,5-trimethoxyphenyl)acryloyl]piperazine-1-carboxylate (12n)

A light yellow solid, yield:52.7%, mp: 137.3–138.2 °C, ^1^H NMR (600 MHz, CDCl_3_) *δ*: 7.58 (d, *J* = 15.3 Hz, 1H, =CH), 6.75–6.70 (m, 3H, ArH and = CH), 3.90–3.83 (m, 9H, OCH_3_), 3.69–3.64 (m, 4H, NCH_2_), 3.47 (s, 4H, NCH_2_), 1.46 (s, 9H, C (CH_3_)_3_).

### *Tert*-butyl 4-[(*E*)-3-(furan-2-yl)acryloyl]piperazine-1-carboxylate (12o)

A brown yellow solid, yield: 42.3%, mp: 103.7–104.7 °C, ^1^H NMR (600 MHz, CDCl_3_) *δ*: 7.45 (d, *J* = 15.1 Hz, 1H, =CH), 7.42 (d, *J* = 1.4 Hz, 1H, furan-H), 6.75 (d, *J* = 15.1 Hz, 1H, =CH), 6.53 (d, *J* = 3.4 Hz, 1H, furan-H), 6.43 (dd, *J* = 3.4, 1.8 Hz, 1H, furan-H), 3.63 (t, *J* = 9.6 Hz, 4H, NCH_2_), 3.45 (t, *J* = 7.3 Hz, 4H, NCH_2_), 1.45 (s, 9H, C (CH_3_)_3_).

### General procedure for preparation of target compounds 5a–o

To a solution of **9** (1.2 g, 4.1 mmol) in DCM (30 mL) were added N,N-Diisopropylethylamine (DIPEA, 1.1 mL, 6.2 mmol) and hexafluorophosphate benzotriazole tetramethyl uranium (HBTU, 2.4 g, 6.2 mmol), followed by an appropriate solution of **13a–o** (4.1 mmol) in DCM. The reaction mixture was stirred at room temperature for 18 h and was then poured into water (40 mL), which was followed by extraction with DCM (3 × 20 mL). The combined organic extracts were dried over Na_2_SO_4_ and evaporated under reduced pressure to give crude product. The pure product was isolated by silica gel column chromatography as a white or yellow powder. Data of these compounds were shown below:

### (E)-4-{[3-[4-(3-phenylacryloyl) piperazine-1-carbonyl]-4-fluorophenyl]methyl}-2H-phthalazin-1-one (5a)

A white solid, yield: 18.3%, mp: 270.8–272.4 °C, ^1^H NMR (600 MHz, DMSO-d_6_) *δ*: 12.57 (s, 1H), 8.23 (d, *J* = 7.8 Hz, 1H), 7.95 (d, *J* = 8.1 Hz, 1H), 7.87 (t, *J* = 8.2 Hz, 1H), 7.80 (s, 1H), 7.67–7.70 (m, 2H), 7.48 (d, *J* = 15.4 Hz, 1H), 7.47–7.35 (m, 4H), 7.29–7.14 (m, 3H), 4.31 (s, 2H), 3.77–3.48 (m, 6H), 3.20 (s, 2H); ^13^C NMR (150 MHz, DMSO-d_6_) *δ*: 165.06, 164.49, 159.82, 157.63, 156.01, 145.27, 142.29, 135.49, 135.28, 133.94, 132.20, 132.00, 130.07, 129.53, 129.41, 129.20, 128.48, 128.34, 126.51, 125.89, 124.06, 123.92, 118.46, 116.46, 47.36, 46.77, 45.61, 45.04, 36.88; IR (KBr, cm^−1^): 3435, 3011, 2911, 1649, 1606, 1217, 769; ESI-MS: 495.8 [M − H]^−^.

### (E)-4-{[3-[4–(3-(3,4-dimethoxyphenyl)acryloyl) piperazine-1-carbonyl]-4-fluorophenyl]methyl}-2H-phthalazin-1-one (5b)

A white solid, yield: 20.3%, mp: 197.9–199.3 °C, ^1^H NMR (600 MHz, DMSO-d_6_) *δ*: 12.57 (s, 1H), 8.23 (d, *J* = 7.8 Hz, 1H), 7.95 (d, *J* = 8.0 Hz, 1H), 7.87 (t, *J* = 7.6 Hz, 1H), 7.80 (t, *J* = 7.3 Hz, 1H), 7.45–7.30 (m, 4H), 7.24–7.19 (m, 3H), 6.94 (d, *J* = 8.3 Hz, 1H), 4.31 (s, 2H), 3.79–3.76(m, 7H), 3.63–3.48 (m, 5H), 3.20(s, 2H); ^13^C NMR (150 MHz, DMSO-d_6_) *δ*: 165.35, 164.48, 159.83, 157.63, 156.01, 150.79, 149.36, 145.28, 142.72, 135.27, 133.93, 132.25, 131.99, 129.53, 129.42, 128.34, 128.30, 126.51, 125.88, 123.94, 122.87, 116.43, 115.69, 111.95, 110.80, 56.13, 55.98, 47.41, 46.87, 45.62, 45.07, 36.88; IR (KBr, cm^−1^): 3443, 3010, 2909, 1638, 1593, 1261, 835; ESI-MS: 555.8 [M − H]^−^.

### (E)-4-{[3-[4–(3-(4-methoxyphenyl) acryloyl) piperazine-1-carbonyl]-4-fluorophenyl] methyl} -2H-phthalazin-1-one (5c)

A white solid, yield: 19.7%, mp: 212.5–213.2 °C, ^1^H NMR (600 MHz, DMSO-d_6_) *δ*: 12.56 (s, 1H), 8.23 (d, *J* = 7.8 Hz, 1H), 7.94 (d, *J* = 8.0 Hz, 1H), 7.87 (td, *J* = 7.7, 1.3 Hz, 1H), 7.80–7.69 (m, 3H), 7.44–7.33 (m, 4H), 7.20–7.17 (m, 2H), 7.04 (d, *J* = 8.3 Hz, 1H), 6.95 (t, *J* = 7.4 Hz, 1H), 4.31 (s, 2H), 3.82 (s, 3H), 3.62–3.47 (m, 6H), 3.20 (s, 2H); ^13^C NMR (150 MHz, DMSO-d_6_) *δ*: 165.34, 164.49, 159.83, 157.89, 157.63, 156.01, 145.27, 136.91, 135.27, 133.93, 132.24, 132.19, 131.58, 129.53, 128.34, 126.51, 125.88, 123.80, 120.98, 118.20, 116.43, 112.05, 56.03, 47.34, 46.80, 45.58, 45.12, 36.88; IR (KBr, cm^−1^): 3455, 3014, 2911, 1660, 1633, 1269, 847; ESI-MS: 526.1 [M − H]^−^.

### (E)-4-{[3-[4-(3-(4-methylphenyl) acryloyl) piperazine-1-carbonyl]-4-fluorophenyl] methyl} -2H-phthalazin-1-one (5d)

A white solid, yield: 15.8%, mp: 245.6–245.9 °C, ^1^H NMR (600 MHz, DMSO-d_6_) *δ*: 12.56 (s, 1H), 8.24 (d, *J* = 7.8 Hz, 1H), 7.94 (d, *J* = 8.04 Hz, 1H), 7.90 (t, *J* = 11.94 Hz, 1H), 7.87 (t, *J* = 7.38 Hz, 1H), 7.59–7.56 (m, 2H), 7.48–7.36 (m, 3H), 7.23–7.19 (m, 4H), 4.31 (s, 2H), 3.76–3.47 (m, 6H), 3.21–3.18 (m, 2H), 2.30 (s, 3H); ^12^C NMR (150 MHz, DMSO-d_6_) *δ*: 165.18, 164.48, 159.82, 157.63, 156.01, 145.27, 142.33, 139.86, 135.28, 133.93, 132.75, 132.20, 131.99, 129.80, 129.44, 128.47, 128.34, 126.51, 125.88, 124.06, 117.27, 116.43, 47.37, 46.77, 45.59, 45.15, 36.88, 21.41; IR (KBr, cm^−1^): 3433, 3111, 2906, 1649, 1606, 1277, 847; ESI-MS: 509.8 [M − H]^−^.

### (E)-4-{[3-[4-(3-(2-methoxyphenyl) acryloyl) piperazine-1-carbonyl]-4-fluorophenyl] methyl}-2H-phthalazin-1-one (5e)

A white solid, yield: 15.2%, mp: 243.8–244.0 °C, ^1^H NMR (600MHz, DMSO-d_6_) *δ*: 12.56 (s, 1H), 8.23 (d, *J* = 7.7 Hz, 1H), 7.94 (d, *J* = 8.0 Hz, 1H), 7.89–7.84 (m, 1H), 7.83–7.62 (m, 3H), 7.43–7.33 (m, 3H), 7.23–7.18 (m, 2H), 7.03 (d, *J* = 8.2 Hz, 1H), 6.95 (t, *J* = 7.5 Hz, 1H), 4.30 (s, 2H), 3.82 (s, 3H), 3.73–3.47 (m, 6H), 3.23–3.18 (m, 2H); ^13^C NMR (150 MHz, DMSO-d_6_) *δ*: 165.35, 164.49, 159.83, 157.89, 157.71, 156.00, 145.28, 136.88, 135.27, 133.93, 132.19, 131.98, 131.58, 129.53, 129.42, 128.34, 126.51, 125.87, 123.94, 123.80, 120.97, 118.20, 116.44, 112.05, 56.03, 47.32, 46.77, 45.59, 45.11, 36.88; IR (KBr, cm^−1^): 3434, 3012, 2908, 1661, 1634, 1268, 980; ESI-MS: 525.4 [M − H]^−^.

### (E)-4-{[3-[4–(3-(2,5-dimethoxyphenyl) acryloyl) piperazine-1-carbonyl]-4-fluorophenyl] methyl} -2H-phthalazin-1-one (5f)

A white solid, yield: 23.9%, mp: 133.9–134.6 °C, ^1^H NMR (600 MHz, DMSO-d_6_) *δ*: 12.56 (s, 1H), 8.23 (d, *J* = 7.9 Hz, 1H), 7.94 (d, *J* = 8.1 Hz, 1H), 7.86 (t, *J* = 7.6 Hz, 1H), 7.82–7.71 (m, 2H), 7.43–7.27 (m, 3H), 7.24–7.10 (m, 2H), 6.97–6.91 (m, 2H), 4.30 (s, 2H), 3.76(s, 3H), 3.73(s, 3H) 3.62–3.47 (m, 6H), 3.23–3.19 (m, 2H); ^13^C NMR (150 MHz, DMSO-d_6_) *δ*: 165.28, 164.46, 159.81, 157.61, 153.63, 152.28, 145.26, 136.70, 135.25, 133.92, 132.23, 131.97, 129.51, 128.32, 126.50, 125.87, 124.41, 123.94, 118.41, 116.97, 116.41, 113.28, 112.97, 56.50, 54.03, 47.38, 46.74, 45.57, 45.09, 36.86; IR (KBr, cm^−1^): 3432, 3109, 2909, 1641, 1565, 1224, 844; ESI-MS: 556.6 [M − H]^−^.

### (E)-4-{[3-[4–(3-(4-fluorophenyl) acryloyl) piperazine-1-carbonyl]-4-fluorophenyl] methyl} -2H -phthalazin-1-one (5g)

A white solid, yield: 15.1%, mp: 272.7–272.9 °C, ^1^H NMR (600 MHz, DMSO-d_6_) *δ*: 12.56 (s, 1H), 8.23 (d, *J* = 8.9 Hz, 1H), 7.94 (d, *J* = 8.0 Hz, 1H), 7.86 (t, *J* = 7.6 Hz, 1H), 7.84–7.67 (m, 3H), 7.47 (d, *J* = 15.3 Hz, 1H), 7.4–7.31 (m, 2H), 7.27–7.07 (m, 4H), 4.30 (s, 2H), 3.83–3.45 (m, 6H), 3.24–3.17 (m, 2H); ^13^C NMR (150 MHz, DMSO-d6) *δ*: 164.87, 164.49, 159.83, 157.64, 156.01, 145.27, 140.97, 134.81, 133.93, 132.14, 132.00, 130.47, 129.53, 129.43, 128.34, 126.51, 125.88, 124.04, 123.92, 123.28, 119.39, 116.29, 47.37, 46.77, 45.69, 45.12, 36.88; IR (KBr, cm^−1^): 3435, 3109, 2908, 1654, 1276, 839; ESI-MS: 513.2 [M − H]^−^.

### (E)-4-{[3-[4–(3-(4-trifluoromethylphenyl) acryloyl) piperazine-1-carbonyl]-4-fluorophenyl] methyl} -2H -phthalazin-1-one (5h)

A light yellow solid, yield: 19.3%, mp: 213.6–214.0 °C, ^1^H NMR (600 MHz, DMSO-d_6_) *δ*: 12.56 (s, 1H), 8.23 (d, *J* = 7.8 Hz, 1H), 7.96–7.84 (m, 3H), 7.83–7.76 (m, 2H), 7.73 (d, *J* = 8.3 Hz, 2H), 7.53 (d, *J* = 15.4 Hz, 1H), 7.47–7.29 (m, 3H), 7.22 (t, *J* = 9.0 Hz, 1H), 4.31 (s, 2H), 3.82–3.46 (m, 6H), 3.22–3.17 (m, 2H); ^13^C NMR (150 MHz, DMSO-d_6_) *δ*: 164.66, 164.52, 159.83, 157.43, 155.87, 145.27, 140.44, 139.56, 133.93, 131.99, 129.85, 129.53, 129.44, 129.10, 128.34, 126.51, 126.03, 125.88, 121.50, 116.39, 47.34, 46.79, 45.64, 45.18, 36.88; IR (KBr, cm^−1^): 3453, 3069, 2919, 1658, 1614, 1280, 981; ESI-MS: 563.2 [M − H]^−^.

### (E)-4-{[3-[4–(3-(3-fluorophenyl) acryloyl) piperazine-1-carbonyl]-4-fluorophenyl] methyl} -2H -phthalazin-1-one (5i)

A white solid, yield: 16.5%, mp: 262.7–263.5 °C, ^1^H NMR (600MHz, DMSO-d_6_) *δ*: 12.56 (s, 1H), 8.23 (d, *J* = 7.8 Hz, 1H), 7.94 (d, *J* = 8.1 Hz, 1H), 7.89–7.84 (m, 1H), 7.82–7.74 (m, 1H), 7.66–7.58 (m, 1H), 7.53–7.31 (m, 5H), 7.23–7.17 (m, 3H), 4.31 (s, 2H), 3.77–3.47 (m, 6H), 3.22–3.17(m 2H); ^13^C NMR (150 MHz, DMSO-d_6_) *δ*: 164.79, 164.46, 163.82, 162.16, 159.82, 145.27, 140.91, 138.13, 135.27, 133.94, 132.26, 132.00, 131.17, 129.53, 129.43, 128.34, 126.51, 125.89, 120.12, 116.80, 116.66, 114.58, 114.40, 47.38, 46.81, 45.62, 45.13, 36.88; IR (KBr, cm^−1^): 3435, 3109, 2907, 1651, 1609, 1251, 974; ESI-MS: 513.2 [M − H]^−^.

### (E)-4-{[3-[4–(3-(4-chlorophenyl) acryloyl) piperazine-1-carbonyl]-4-fluorophenyl] methyl} -2H -phthalazin-1-one (5j)

A white solid, yield: 17.2%, mp: 239.2–240.4 °C, ^1^H NMR (600 MHz, DMSO-d_6_) *δ*: 12.56 (s, 1H), 8.23 (d, *J* = 7.3 Hz, 1H), 7.94 (d, *J* = 8.1 Hz, 1H), 7.89–7.84 (m, 1H), 7.80–7.70(m, 3H), 7.49–7.40 (m, 4H), 7.38–7.26 (m, 2H), 7.21 (t, *J* = 9.0 Hz, 1H), 4.30 (s, 2H), 3.81–3.43 (m, 6H), 3.22–3.17 (m, 2H); ^13^C NMR (150 MHz, DMSO-d_6_) *δ*: 164.88, 164.50, 159.83, 157.63, 156.01, 145.27, 140.88, 135.26, 134.51, 134.47, 133.92, 132.27, 132.22, 131.98, 130.21, 129.53, 129.21, 128.34, 126.51, 125.87, 124.01, 119.33, 116.29, 47.31, 46.76, 45.63, 45.09, 36.89; IR (KBr, cm^−1^): 3435, 3108, 2908, 1657, 1492, 1273, 842; ESI-MS: 529.2 [M − H]^−^.

### (E)-4-{[3-[4–(3-(4-bromophenyl) acryloyl) piperazine-1-carbonyl]-4-fluorophenyl] methyl} -2H -phthalazin-1-one (5k)

A white solid, yield: 18.2%, mp: 225.7–226.0 °C, ^1^H NMR (600 MHz, DMSO-d_6_) *δ*: 12.56 (s, 1H), 8.23 (d, *J* = 7.74 Hz, 1H), 7.94 (d, *J* = 8.0 Hz, 1H), 7.88–7.84 (m, 1H), 7.80 (t, *J* = 7.3 Hz, 1H), 7.67–7.62 (m, 2H), 7.57 (d, *J* = 8.46 Hz, 2H), 7.46–7.18 (m, 5H), 4.30 (s, 2H), 3.75–3.47 (m, 6H), 3.19–3.13 (m, 2H); ^13^C NMR (150 MHz, DMSO-d_6_) *δ*: 164.87, 164.50, 159.83, 157.64, 156.01, 145.27, 140.97, 135.27, 134.81, 133.93, 132.27, 132.22, 132.14, 131.99, 130.47, 129.53, 129.41, 128.34, 126.51, 125.87, 123.92, 123.28, 119.39, 116.29, 47.41, 46.53, 45.69, 45.12, 36.89; IR (KBr, cm^−1^): 3461, 3064, 2915, 1657, 1612, 1359, 1286, 1224; ESI-MS: 573.1 [M − H]^−^.

### (E)-4-{[3-[4–(3-(3-nitrophenyl) acryloyl) piperazine-1-carbonyl]-4-fluorophenyl] methyl} -2H -phthalazin-1-one (5l)

A white solid, yield: 16.9%, mp: 266.8–268.2 °C, ^1^H NMR (600 MHz, DMSO-d_6_) *δ*: 12.56 (s, 1H), 8.58 (d, *J* = 30.2 Hz, 1H), 8.23 (d, *J* = 7.8 Hz, 1H), 8.19–8.08 (m, 2H), 7.94 (d, *J* = 8.1 Hz, 1H), 7.86 (td, *J* = 7.7, 1.3 Hz, 1H), 7.81–7.80 (m, 1H), 7.67 (t, *J* = 8.0 Hz, 1H), 7.59 (d, *J* = 15.4 Hz, 1H), 7.50–7.53 (m, 1H), 7.43–7.35 (m, 3H), 7.22 (t, *J* = 9.0 Hz, 1H), 4.31 (s, 2H), 3.80–3.49 (m, 6H), 3.21 (s, 2H); ^13^C NMR (150 MHz, DMSO-d_6_) *δ*: 164.61, 159.82, 157.72, 156.47, 148.80, 145.26, 139.92, 137.41, 135.29, 135.00, 133.92, 132.22, 131.98, 130.66, 129.52, 129.40, 128.34, 126.51, 125.87, 124.29, 122.55, 121.61, 47.74, 46.76, 45.67, 45.10, 36.89; IR (KBr, cm^−1^): 3436, 3109, 2907, 1645, 1596, 1277, 841; ESI-MS: 540.9 [M − H]^−^.

### (E)-4-{[3-[4–(3-(4-nitrophenyl) acryloyl) piperazine-1-carbonyl]-4-fluorophenyl] methyl} -2H -phthalazin-1-one (5m)

A white solid, yield: 17.3%, mp: 224.0–224.5 °C, ^1^H NMR (600 MHz, DMSO-d_6_) *δ*: 12.59 (s, 1H), 8.24 (t, *J* = 8.3 Hz, 3H), 8.01–7.95 (m, 3H), 7.89–7.82 (m, 2H), 7.58 (d, *J* = 15.4 Hz, 1H), 7.45–7.37 (m, 3H), 7.23 (t, *J* = 8.94 Hz, 1H), 4.32 (s, 2H), 3.81–3.51 (m, 6H), 3.27–3.18 (m, 2H); ^13^C NMR (150 MHz, DMSO-d_6_) *δ*: 164.45, 159.83, 157.59, 156.02, 148.01,145.28, 142.10, 139.74, 135.29, 133.94, 132.26, 132.01, 129.52, 129.44, 128.33, 126.52, 125.89, 124.32, 123.99, 123.07, 116.47, 116.33, 47.33, 46.73, 45.68, 45.20, 36.88; IR (KBr, cm^−1^): 3436, 3072, 2926, 1766, 1648, 1274, 842; ESI-MS: 539.7 [M − H]^−^.

### (E)-4-{[3-[4–(3-(3,4,5-trimethoxyphenyl) acryloyl) piperazine-1-carbonyl]-4-fluorophenyl] methyl} -2H -phthalazin-1-one (5n)

A white solid, yield: 26.8%, mp: 235.3–236.2 °C, ^1^H NMR (600 MHz, DMSO-d_6_) *δ*: 12.56 (s, 1H), 8.23 (d, *J* = 7.1 Hz, 1H), 7.94 (d, *J* = 8.1 Hz, 1H), 7.90–7.84 (m, 1H), 7.80 (d, *J* = 7.7 Hz, 1H), 7.47–7.34 (m, 3H), 7.22 (t, *J* = 9.0 Hz, 2H), 7.03–7.00 (m, 2H), 4.31 (s, 2H), 3.79 (s, 7H), 3.71–3.46 (m, 9H), 3.22–3.16 (m, 2H); ^13^C NMR (150 MHz, DMSO-d_6_) *δ*: 165.13, 164.46, 159.81, 157.62, 155.99, 153.46, 145.26, 142.82, 139.31, 135.27, 133.93, 132.25, 132.19, 131.98, 131.04, 129.51, 128.32, 126.50, 125.87, 124.03, 123.92, 117.37, 116.41, 106.13, 60.52, 56.50, 54.04, 47.32, 46.80, 45.57, 44.90, 36.86; IR (KBr, cm^−1^): 3435, 3109, 2908, 1642, 1270, 843; ESI-MS: 586.2 [M − H]^−^.

### (E)-4-{[3-[4-(3-(furan-2-yl) acryloyl) piperazine-1-carbonyl]-4-fluorophenyl] methyl} -2H -phthalazin-1-one (5o)

A light yellow solid, yield: 17.8%, mp: 152.5–155.0 °C, ^1^H NMR (600 MHz, DMSO-d_6_) *δ*: 12.56 (s, 1H), 8.23 (d, *J* = 7.8 Hz, 1H), 7.93 (d, *J* = 8.0 Hz, 1H), 7.86 (t, *J* = 7.1 Hz, 1H), 7.80 (td, *J* = 7.5, 0.78 Hz, 1H), 7.60 (d, *J* = 1.3 Hz, 1H), 7.43–7.31 (m, 3H), 7.21 (t, *J* = 9.0 Hz, 1H), 6.92–6.83 (m, 2H), 6.57 (dd, *J* = 3.2, 1.8 Hz, 1H), 4.30 (s, 2H), 3.67–3.47 (m, 6H), 3.19–3.10 (m, 2H); ^13^C NMR (150 MHz, DMSO-d_6_) *δ*: 164.69, 164.48, 159.83, 157.63, 156.01, 151.57, 145.39, 145.28, 135.25, 133.94, 132.25, 132.20, 131.99, 129.57, 129.53, 129.44, 129.42, 128.33, 126.51, 125.89, 124.04, 116.44, 116.30, 114.65, 112.98, 47.02, 46.59, 45.55, 45.18, 36.88; IR (KBr, cm^−1^): 3431, 3102, 2908, 2865, 1645, 1275, 844; ESI-MS: 485.5 [M − H]^+^.

## Biological activity

### PARP inhibition assay

PARP enzyme inhibition was measured using an HT F Homogeneous 96-well PARP Inhibition Assay Kit (Trevigen, Cat# 4690–096-K, Gaithersburg, USA), according to the manufacturer’s protocol. The various synthesized compounds were dissolved in DMSO and then serially diluted to the required concentrations with distilled water, keeping the final concentration of DMSO lower than 1%. Olaparib was used as positive control. Fluorescence values under the condition of excitation wavelength (544 nm) and emission wavelength (590 nm) were measured using a multiwell spectrophotometer (Molecular Devices SpectraMax M5 microplate reader, Careforde, Chicago, USA). Then, the standard curve was drawn and the inhibition rate of each test compound was calculated. IC_50_ value of each compound was calculated using GraphPad Prism 6 software according to the above results.

### MTT assay for cell growth inhibition

The anti-proliferative activities of synthesized compounds were assessed against breast cancer cell line MDA-MB-436 by the 3-(4, 5-Dimethylthiazol-2-yl)-2,5-diphenyltetrazolium bromide (MTT) assay. The MDA-MB-436 cell was purchased from Chinese Academy of Sciences. Briefly, cells were seeded in 96-well plates at a density of 4 × 10^4^ cells per mL in Hyclone RPMI medium supplemented with 10% foetal bovine serum and then treated with different concentrations of synthesized compounds in DMSO. Olaparib was used as the positive control. After treated with compounds for 72 h, cells were incubated with 20 μL of 5 mg/mL MTT for 4 h. The culture medium was removed, and 150 μL DMSO was added to dissolve the formazan crystals. Wells containing only media were used for background correction. The optical density was measured spectrophotometrically at 490 nm. EC_50_ values were calculated using GraphPad Prism 6 software. Data were expressed as mean ± SEM.

### Anti-cholinesterase activity assays

The acetylcholinesterase (eeAChE, from the electric eel), butyrylcholinesterase (eqBChE, from equine serum), 5, 5’-dithiobis-2-nitrobenzoic acid (Ellman's reagent, DTNB), S-butyrylthiocholine iodide (BTCI), acetylthiocholine iodide (ATCI), and Donepezil hydrochloride and Neoeserine methyl sulfate were purchased from Sigma-Aldrich.

The capacity of the test compounds **5a–o** to inhibit the AChE and BChE activities were assessed by the Ellman's method in 96-well plates with slight modification^31^. The Donepezil and Neoeserine were used as reference drugs. The test compounds were dissolved in a minimum volume of DMSO (1%) and were diluted using sodium phosphate buffer (0.1 M, pH 7.4). A reaction mixture containing 20 μL sodium phosphate buffer, 100 μL DTMB (1 mM) and 40 μL AChE (0.2 U/Ml)/BChE (0.3 U/mL) was incubated with 20 μL of various concentrations of test compounds at 37 °C for 15 min followed by the addition of the substrate (20 μL) acetylthiocholine iodide (1 mM) or S-butyrylthiocholine iodide (1 mM) and incubation for another 3 min. The activities were determined by using a Varioskan Flash Multimode Reader (Thermo, Miami, FL, USA) at 412 nm. The inhibition per cent was calculated by the following expression: (1-Ai/Ac) × 100, where Ai and Ac were the absorbance obtained for AChE/BChE in the presence and absence of inhibitors, respectively. The concentration of compound producing 50% of enzyme activity inhibition (IC_50_) was calculated by nonlinear regression analysis of the response–concentration (log) curve, using the Graph Pad Prism program package (Graph Pad Software; San Diego, CA). Results are expressed as the mean ± SEM of at least three different experiments performed in triplicate.

### Molecular docking

Molecular docking studies were performed using the AutoDock Vina 1.1.2^32^ along with the AutoDockTools 1.5.6 (ADT) and AutoGridFR 1.0. The structures of compounds **5c**, **5i**, **5l** were drawn and then subjected to energy minimization with the molecular mechanics (MM2) using the ChemBioOffice 13.0 (CambridgeSoft, PerkinElmer Inc., MA, USA). The ligands were further processed and saved in the pdbqt format for docking by ADT program. The X-ray crystallographic structures of PARP-1 (PDB Code 5DS3)^33^ and hBChE (PDB Code 5NN0)^34^ were obtained from the online PDB database (RCSB) (http://www.rcsb.org). Both receptors were firstly prepared using the Acceryls Discovery Studio 2017 R2 (Accelrys Inc., San Diego, CA, USA) involving removal of water molecules, non-bonded inhibitors and cofactors. Subsequently, both proteins were edited using the ADT program to add the polar hydrogen atoms and Kollman charges as means to rigidify protein structures and also saved in the same pdbqt format as the ligands **5c**, **5i**, **5l**. The torsions for ligands and receptors were set to be rotatable and rigid respectively. The AutoGridFR software was applied to determine the binding pocket and the grid coordinate^35^. After that, docking was performed using the default settings of Vina and only the minimum predicted Gibbs binding energy pose was considered for each case. Analysis and visualization of the docking results were done using the PyMOL version 1.78 for 3D diagrams (The PyMOL Molecular Graphics System, Version 1.6. Schrödinger, LLC.) and the LigPlot ^+^ version 1.45^36^ for 2D ones.

## Results and discussion

### Chemistry

**Scheme 1.**
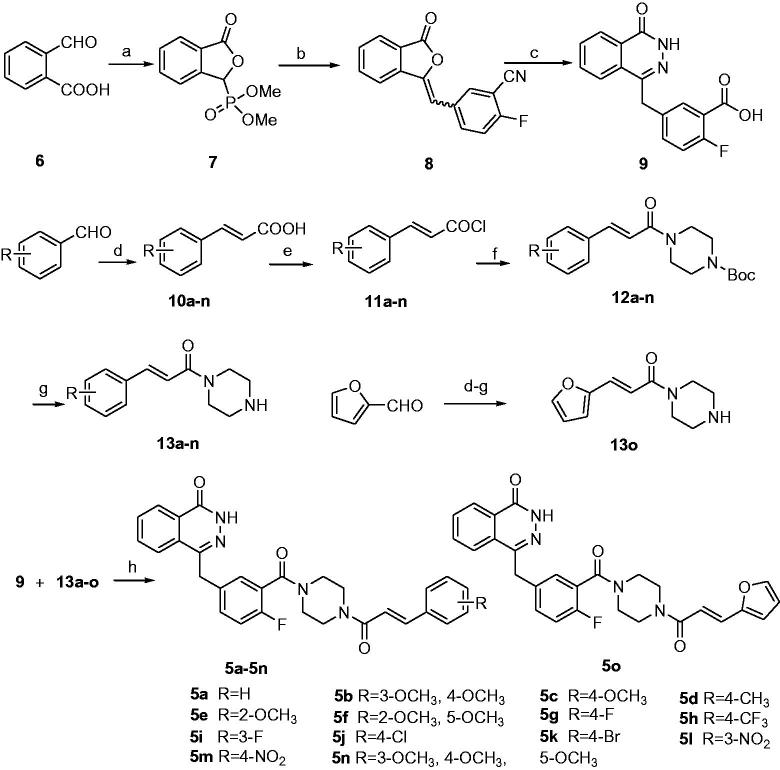
Synthesis route for target compounds. Reactions and conditions: (a) dimethyl phosphite, 100 °C, 8 h, 62.3%; (b) 2-fluoro-5-formylbenzonitrile, Et_3_N, anhydrous THF, 20 °C, 16 h, 92.6%; (c) (i) H_2_O, 10mol/l NaOH, 90 °C, 1 h, (ii) NH_2_NH_2_·H_2_O, 70 °C, 18 h, 4 mol/l HCl, 93.7%; (d) propane diacid, NH_4_Ac, 85 °C, 5 h, 50.0–80.0%; (e) oxalyl chloride, DMF, 20 °C, 1.5 h; (f) *Tert*-butoxycarbonylpiperazine, Et_3_N, DCM, 20 °C, 12 h, 40.0–70.0%; (g) TFA, DCM, 20 °C, 8 h, 50.0–80.0%; (h) HBTU/DIPEA, DCM, 20 °C, 18 h, 15.7–26.8%;

The synthetic route for the desired compounds **5a–o** is described in Scheme 1. 2-Formyl benzoic acid **6** was used as starting material for the preparation of the key intermediate **9**, which firstly reacted with dimethyl phosphate to afford the dimethyl (1, 3-dihydro-3-oxoisobenzofuran-1-yl)-1-phosphonate **7**, and then, the Wittig reaction of **7** with 2-fluoro-5-formylbenzonitrile was carried out to give compound **8**. The benzoic acid derivative **9** was finally obtained after hydrolysis of **8**. Condensations of propanedioic acid with various substituted benzaldehydes or furan-2-carbaldehyde followed by decarboxylation reactions furnished compounds **10a–o**. Treatment of **10a–o** with oxalyl chloride followed by 1-boc-piperazine gave amides **12a–o**, which were subsequently deprotected to provide compounds **13a–o**. Finally, intermediates **9** reacted with **13a–o** to afford the target compounds **5a–o**.

The structures of newly synthesized target compounds **5a–o** were confirmed by various spectral techniques. The IR spectra of **5a**–**o** exhibited absorption bands at around 3400 cm^−1^ which was assigned to NH group stretching vibration. In the ^1^H NMR spectra of **5a–o** the N–H appeared at about 12.56 ppm. In the ^1^H NMR spectra of **12a**–**o,** the vinylic protons appeared as doublet at 7.40–7.90. The large coupling constant (*J* = 15.4 Hz) of the vinylic protons confirmed the trans configuration.

### Biology

The *in vitro* PARP-1 enzyme inhibitory activities of all the target compounds were firstly screened at the fixed concentration of 0.5 μM and then, the ones with inhibitory rates ≥80% were selected to further determine their IC_50_ value. The results are summarized in Table 1. It showed that all compounds possessed PARP-1 inhibitory activities, but none of them were more potent than Olaparib. **5l** and **5m** exhibited more potent inhibitory effects on PARP-1 than the other compounds, which might attribute to the nitro group on the phenyl ring. Introduction of steric groups into the phenyl ring of aromatic propylene moiety, e.g. the dimethoxy in **5b**, **5f** or the trimethoxy group in **5n**, decreased the inhibitory potency; nevertheless, the less steric group in **5o** with a furyl group also resulted in decreased activity. The cellar inhibitory potencies of all target compounds were tested against breast cancer cell line MDA-MB-436 by MTT assay and the results are shown in Table 2. It indicated that **5c**, **5g**, **5h**, **5i**, **5l** and **5m** had similar effects, among which **5l** exhibited the most potent inhibitory activity. On the cellular level, incorporation groups containing fluorine (**5g**, **5h**, **5i**) helped to increase potency of target compounds perhaps improving bioavailability.

**Table 1.** PARP-1 inhibitory activity of compounds **5a–5o**.

Compd.	Inhibition rate (%)^a^	IC_50_ (nM)^b^	Compd.	Inhibition rate (%)^a^	IC_50_ (nM)^b^
**5a**	75.21 ± 4.84%	ND^c^	**5i**	88.23 ± 5.24%	267.22 ± 9.32
**5b**	77.23 ± 3.89%	ND	**5j**	75.45 ± 4.54%	ND
**5c**	89.23 ± 10.64%	143.78 ± 5.26	**5k**	43.21 ± 6.54%	ND
**5d**	64.21 ± 4.59%	ND	**5l**	95.72 ± 4.35%	16.10 ± 1.25
**5e**	62.56 ± 3.65%	ND	**5m**	93.15 ± 5.28%	37.90 ± 1.89
**5f**	75.62 ± 3.61%	ND	**5n**	65.96 ± 4.82%	ND
**5g**	85.63 ± 3.69%	396.71 ± 7.01	**5o**	65.23 ± 5.98%	ND
**5h**	89.52 ± 12.58%	294.70 ± 8.25	**Olaparib**	98.20 ± 3.59%	8.20 ± 1.06

^a^Data were expressed as mean ± SD, measured at the *conc.* of 0.5 μM, *n*= 3.

^b^Data were expressed as mean ± SD, *n*= 3(*p*<.05); ^c^ND: not determined.

**Table 2.** MDA-MB-436 cell inhibitory activities of compounds **5a–5o**.

Compd.	EC_50_ (μM)^a^^,b^	Compd.	EC_50_ (μM)^a^^,b^
**5a**	38.21 ± 2.15	**5i**	14.17 ± 5.21
**5b**	41.23 ± 1.95	**5j**	45.19 ± 1.68
**5c**	14.20 ± 3.16	**5k**	112.01 ± 5.24
**5d**	35.15 ± 3.42	**5l**	11.62 ± 2.15
**5e**	44.23 ± 1.95	**5m**	13.95 ± 1.28
**5f**	30.18 ± 3.25	**5n**	31.25 ± 4.12
**5g**	13.02 ± 2.14	**5o**	32.23 ± 3.58
**5h**	14.83 ± 1.42	**Olaparib**	8.63 ± 1.25

^a^Data are expressed as mean ± SD, *n*= 3. Incubated for 72 h (*p*<.05).

^b^The EC_50_ value was the concentration required to reduce cell proliferation by 50% in single-agent cytotoxicity assay.

In order to explore the potential of being MTDLs for AD, the *in vitro* inhibitory effects of all target compounds on Electrophorus electricus AChE (EeAChE) and equine serum butyrylcholinesterase (eqBChE) were investigated according to the method^31^. Two drugs, Donepezil and Neostigmine, were used as the standards. The results are summarized in Table 3. The IC_50_ values suggested that most of the synthesized compounds exhibited little-to-moderate inhibitory activities against cholinesterases. Furthermore, a clear trend appeared that with exception of compound **5c** all the other compounds showed better inhibition of BChE than AChE. Although the inhibitory potency against AChE of compound **5m** was weaker than Donepezil and Neostigmine, its inhibitory potency against BChE (5.93 μM) is more potent than the two drugs (7.64 and 12.01 μM, respectively). Comparison of 3, 4-dimethanoxy derivative **5b** with 3, 4, 5-trimethanoxy one **5n**, 2-methanoxy compound **5e** with 2, 5-dimethanoxy one **5f**, demonstrated the relatively compact group decreased its inhibitory activities against cholinesterase. Halogen-substituted compounds **5g–k** having F, CF_3_, Cl, Br on the benzene ring appear less active than the unsubstituted compound **5a**. Both compounds **5l** and **5m** with a strong electron-withdrawing nitro group showed the most potent activities among these derivatives.

**Table 3.** AChE and BChE inhibitory activities of compounds **5a–5o**.

Compd.	IC_50_ (μM)^a^		Compd.	IC_50_ (μM)^a^	
	AChE^b^	BChE^b^		AChE	BChE
Donepezil	0.03 ± 0.004	7.64 ± 0.21	Neostigmine	0.04 ± 0.01	12.01 ± 0.45
Olaparib	50.95 ± 0.46	34.36 ± 0.64	**5h**	136.43 ± 12.51	56.76 ± 04.36
**5a**	52.91 ± 3.02	22.40 ± 1.19	**5i**	110.35 ± 9.18	33.39 ± 4.14
**5b**	45.19 ± 1.38	17.75 ± 0.72	**5j**	86.54 ± 5.12	37.08 ± 2.37
**5c**	55.82 ± 0.60	178.84 ± 5.89	**5k**	80.69 ± 4.87	45.75 ± 3.76
**5d**	71.99 ± 3.84	27.81 ± 0.87	**5l**	24.55 ± 1.10	9.16 ± 0.91
**5e**	56.94 ± 4.30	26.40 ± 1.93	**5m**	12.24 ± 0.49	5.93 ± 0.19
**5f**	76.86 ± 4.13	35.72 ± 2.98	**5n**	99.09 ± 2.21	65.22 ± 1.08
**5g**	173.01 ± 29.98	104.63 ± 12.50	**5o**	113.71 ± 14.95	77.57 ± 9.06

^a^Data are expressed as mean ± SD, *n*= 3 (*p* < .05).

^b^AChE from electric eel and BChE from equine serum were used.

The two important cholinesterases, both AChE and BChE, can hydrolyse acetylthiocholine (ACh) in the synaptic gap of neuronal cells, which results in the loss of neurotransmitter and the decline of cognition during AD. The role of BChE for ACh hydrolysis is only of minor impact in healthy brain, but in advanced AD the level of AChE drops dramatically down to 90% and the levels of BChE in certain parts of the brain increase significantly to compensate the loss of neuronal AChE^37^. Besides that, it has been shown that BChE (over-)expression is associated with senile plaques and the transformation of non-fibrillar to fibrillary Aβ plaques. Hence, inhibition of BChE may be more therapeutically valuable than inhibition of AChE in later stages of AD and researches on selective BChE inhibitors are paid wide attention to in recent years^38^^,^^39^. In this study, the synthesized compounds to some degree showed inhibitory selectivity over BChE, although their potencies were not strong enough as nanomolar level, which at least gave a clue to search for new skeleton frame responsible for multifunctional PARP-1 and cholinesterase inhibition.

In biological tests, compound **5l** showed more potent inhibitory activity than **5m** against PARP-1, while **5m** exhibited a little more potent than **5l** against AChE and BChE; meanwhile, these synthesized compounds show inhibitory selectivity over BChE, and taking all together, **5l** was chosen to carry on molecular docking with PARP-1 and BChE proteins to obtain an insight into the molecular level of the ligand binding. Additionally, in order to further investigate the interaction fashion of these new compounds with PARP-1, molecular docking of **5c and 5i** with PARP-1was performed.

## Molecular modelling studies

Docking simulations were performed for **5c, 5**i and **5l** into the PARP-1 (PDB ID: 5DS3) and **5l** into the hBChE (PDB ID: 5NNO) active sites using the AutoDock Vina 1.1.2. The molecular docking protocols were validated through re-docking of the co-crystallized ligands in the vicinity of the PARP-1 and hBChE active site. It reproduced all the key interactions accomplished by the co-crystallized ligand with the key amino acids in the active site as shown in the crystal, indicating the suitability of the used setup for the docking study.

**Figure 2.**
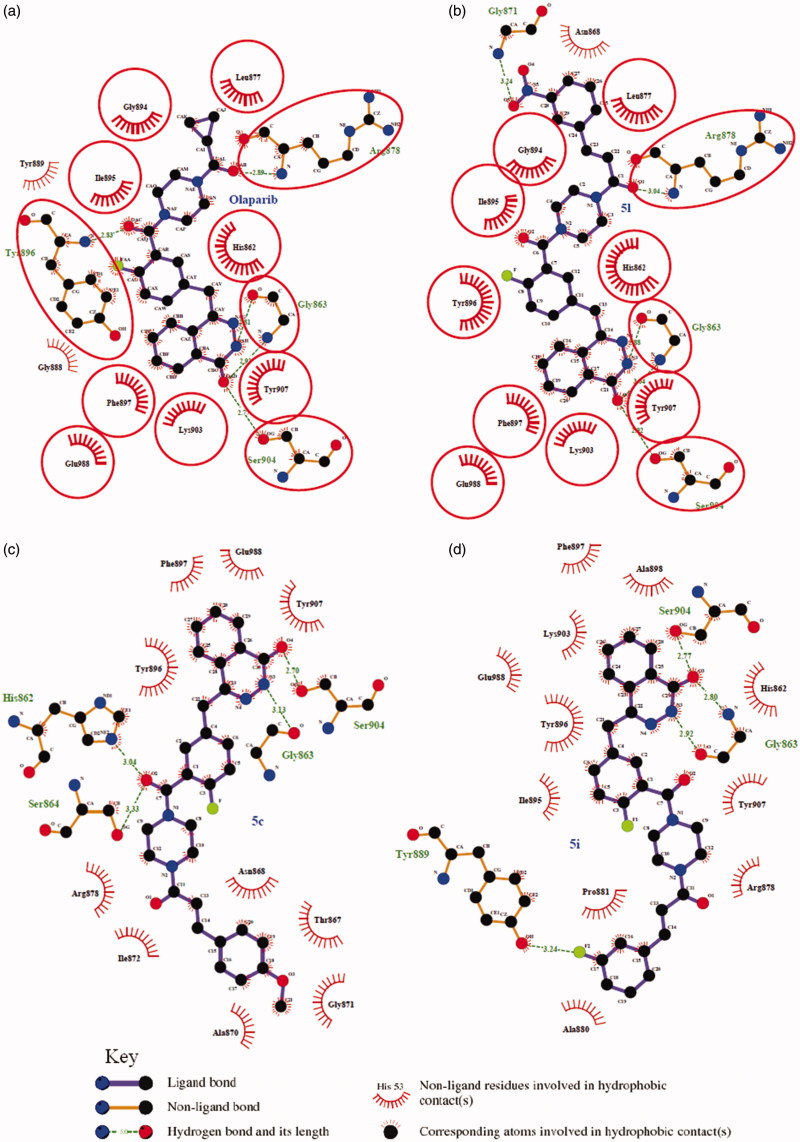
Ligplot images showing the interactions of the Olaparib (a), **5l** (b), **5c** (c) and **5i** (d) with the enzyme PARP-1. The commonly interacting amino acid residues in both interactions were encircled in red circles.

**Figure 3.**
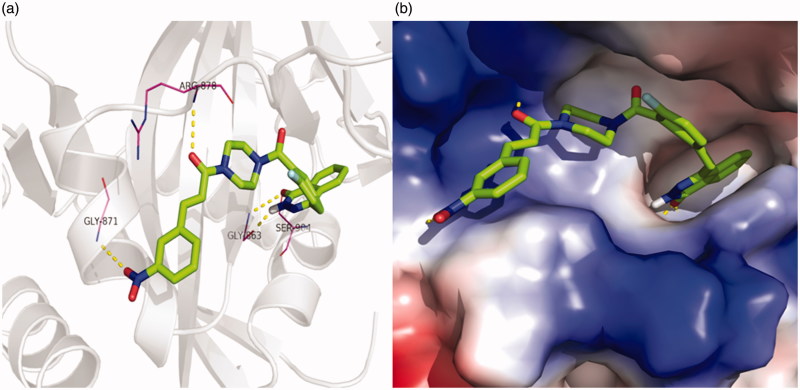
(a) 3D representation of different interactions of compound **5l** with residues in the binding sites of PARP-1. The compound was rendered in green stick model and the residues were rendered in purple sticks. Hydrogen bonds were indicated with yellow dashed lines. (b) A vacuum electrostatics depiction of PARP-1 bound to derivative **5l**, showing protein contact potential. Surface coloring was according to the electrostatic potential: red, white, and blue correspond to negative, neutral, and positive potential, respectively. The vacuum electrostatics/protein contact potential was generated by PyMOL. The **5l** was depicted by sticks.

Regarding PARP-1, molecular docking of **5l** in PARP-1 revealed an optimal binding mode characterized by affinity of the minimum Gibbs binding energy with –11.9 kcal/mol. As illustrated in the 2D diagrams plotted by LigPlot+ (Figure 2), docking simulation of **5l** showed that it fitted into the enzyme active site almost at the same position of co-crystallized ligand Olaparib. Three hydrogen bonds in **5l**-PARP-1 complex were formed in the phthalazinone ring which was well recognized as a scaffold mainly responsible for PARP-1 inhibitory activity (Figures 2(b) and 3(a)): the carbonyl group as H-bond acceptor with the Gly863 A-NH group (3.04 Å) and Ser904 A-OH group (2.92 Å), the –NH group as H-bond donor with Gly863 A-C=O group (2.88 Å). The fourth identical hydrogen bond was established between the C=O group from acryloyl moiety and Arg878 A-NH group (3.04 Å). The newly introduced nitro group formed a hydrogen bond with Gly871 A-NH group (Figures 2(b) and 3(a)). However, another C=O group connected to the piperazine moiety did not form a hydrogen bond with the Tyr896 A-NH group as compared with Olaparib, which perhaps was one of the reasons why **5l** exhibited a less potent effect on PARP-1 inhibition than Olaparib as shown *in vitro* experiments. Besides the interaction of hydrogen bond, compound **5l** formed hydrophobic interactions with residues Tyr907, Lys903, Phe897, Tyr896, His862, Ile895, Gly894, Leu877 and Asn868. The minimum Gibbs binding energies of molecular docking of **5c** and **5i** into PARP-1 were –11.9 and –11.8 kcal/mol respectively. As shown in Figure 2, the phthalazinone rings of both compounds **5c** (Figure 2(c)) and **5d** (Figure 2(d)) formed corresponding two and three hydrogen bonds with PARP-1which were similar with those of **5l** and Olaparib: the carbonyl group as H-bond acceptor with the Ser904 A-OH group or Gly863 A-NH group, the –NH group as H-bond donor with Gly863 A – C=O group. However, within the other moieties of **5c** and **5i** just one position formed hydrogen bond with PARP-1 compared to two positions within those of **5l** and Olaparib. The differences were maybe the reason why **5c** and **5l** exhibited weaker potency than **5l**. On the basis of the docked conformation, the protein contact potential was generated for the compound **5l** (Figure 3(b)) depicting the whole surface of the protein. Red and blue colours showed negative and positive charges, respectively. In this depiction, it was shown that compound **5l** was bound inside a cavity formed at the interface of the enzyme.

The crystal structure of the recombinant human BChE (PDB ID: 5NNO) complexed with ligand 92H has been reported recently^34^. The compound **92H**, N-((1–(2, 3-dihydro-1*H*-inden-2-yl) piperidin-3-yl)methyl)-N-(2-(dimethyl-amino)ethyl)-2-naphthamide, shows high selectivity and an extremely potent inhibitory effect on huBChE with picomolar affinity (IC_50_=1.03 ± 0.04 nM); therefore, 5NN0 was used for docking to analyse the key binding interactions of the synthesized compound **5l** for huBChE inhibition.

**Figure 4.**
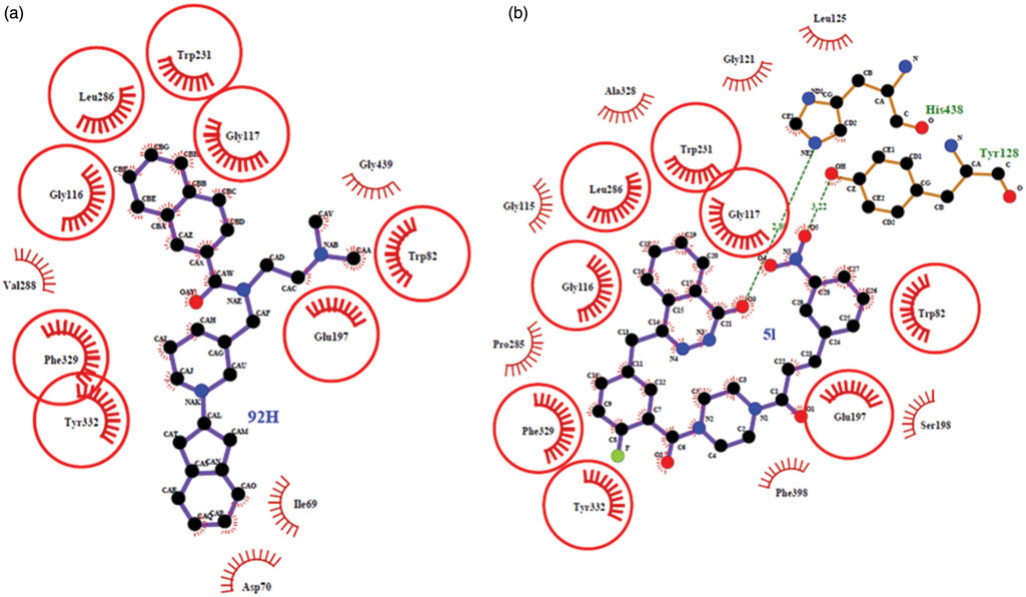
Ligplot image showing the interactions of the **92H** (a) and **5l** (b) with the enzyme huBChE. Arcs with red lines represented amino acid hydrophobic contacts; green dashed lines represents hydrogen bonds. The commonly interacting amino acid residues in both interactions were encircled in red circles.

**Figure 5.**
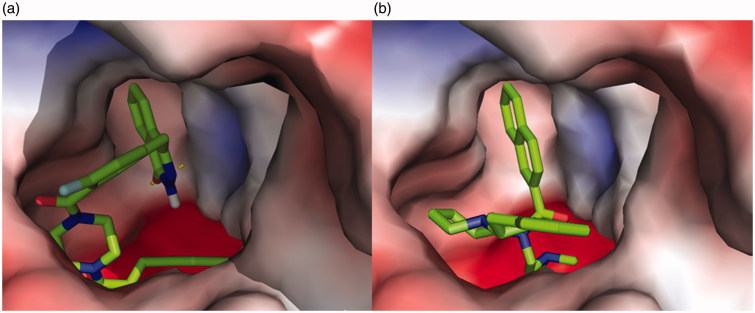
A vacuum electrostatics depiction of huBChE bound to derivative **5l** (a) and reference **92H** (b), showing protein contact potential. Surface colouring was according to the electrostatic potential: red, white, and blue correspond to negative, neutral, and positive potential, respectively. The vacuum electrostatics/protein contact potential was generated by PyMOL. The **5l** and **92H** are depicted by sticks.

The molecular docking of **5l** into huBChE revealed an optimal binding mode characterized by the affinity of the minimum Gibbs binding energy with –13.5 kcal/mol. As illustrated in the 2D diagrams plotted by LigPlot^+^ (Figure 4), docking simulation of **5l** showed that it interacted with the enzyme active site at local positions same as the reference **92H** did. The naphthalene ring of **92H** and the phthalazin-1-one moiety of **5l** all formed hydrophobic interactions with residues Leu286, Gly116, Gly117 and Trp231. The –(CH_2_)_2_NMe_2_ side chain of **92H** formed hydrophobic interactions with residues Trp82 and Glu197 as well as the fluorophenyl group of **5l** did. In particular, the Trp231 and Trp82 were two important residues located, respectively, in the acyl-binding pocket and choline-binding pocket of huBChE with which strong interactions could result in an excellently inhibitory effect on huBChE. In spite of this, there were some differences between **5l** and **92H** about the interactions with residues of huBChE, which perhaps led to a comparatively weaker activity of **5l**. Two hydrogen bonds, the carbonyl group as H-bond acceptor with the His438 A-NH group (2.84 Å) and the nitro group as H-bond acceptor with the Tyr128 –OH group (3.22 Å), were established between **5l** and huBChE, which may be weaken the hydrophobic interaction of **5l** with the key residues Trp82 and Trp231. On the other hand, the 2, 3-dihydro-1*H*-inden moiety of **92H** formed hydrophobic interactions with residues Ile69 and Asp70 while **5l** did not. As shown in the vacuum electrostatics depiction of huBChE bound to **5l** (Figure 5(a)) and **92H** (Figure 5(b)), although both **5l** and **92H** were bound inside a cavity formed at the interface of the enzyme, it was obvious for **5l** to be closely packed. Therefore, it was inferred that the compound with small and flexible groups except for the phthalazin-1-one moiety contributed to enhance its inhibitory activity against huBChE in the pursuit of the PARP-1 and AChE dual-targeted inhibitor.

Generally, the results of respective docking of **5c**, **5i** and **5l** in complexly PARP-1 and **5l** with huBChE indicated that **5l** could interact well with the active sites of PARP-1 protein, but it did not do well with that of huBChE, which provided a basis and valuable information for further studies aimed at identifying inhibitors of both PARP-1 and huBChE.

## Conclusion

Considering that inhibition of PARP-1 has therapeutical values not only for cancer but also for AD, a series of 15 novel Olaparib analogues was synthesized, and their inhibitory activities against the enzymes PARP-1, AChE (from electric eel), BChE (from equine serum) and the cancer cell line MDA-MB-436 were tested. Among these synthesized compounds, **5l** exhibited the most potent inhibitory effects on PARP-1 (16.10 ± 1.25 nM) enzyme and MDA-MB-436 (11.62 ± 2.15 μM) cancer cell, which were just a little weaker than Olaparib. Meanwhile, **5l** displayed moderate BChE inhibitory activity (9.16 ± 0.91 μM) which was stronger than Neostigmine (12.01 ± 0.45 μM) and selectivity for BChE over AChE to some degree. Molecular docking researches implied that the formation of hydrogen bond was necessary for PARP-1 inhibitory potency, but it was not important for BChE inhibitory activity. While keeping phthalazin-1-one unchanged, small and flexible non-polar groups seemed to be conducive to improving inhibitory potency on BChE. Our research gave a clue to search for new agents based on AChE and PARP-1 dual-inhibited activities to treat Alzheimer's disease.

## Supplementary Material

supplemental_file.doc
