# Supplementary material for: Synthesis, preliminarily biological evaluation and molecular docking study of new Olaparib analogues as multifunctional PARP-1 and cholinesterase inhibitors
Source: J Enzyme Inhib Med Chem. 2018 Nov 14;34(1):150–62. doi: 10.1080/14756366.2018.1530224 (PMC6237161; doi:10.1080/14756366.2018.1530224)
Supplement: supplemental_file.doc [file IENZ_A_1530224_SM7857.doc]

**^1^H NMR and ^13^C NMR of target compounds**

***(E)-4-{[3-[4-(3-phenylacryloyl) piperazine-1-carbonyl]-4-fluorophenyl]methyl}-2H-phthalazin-1-one (5a)***


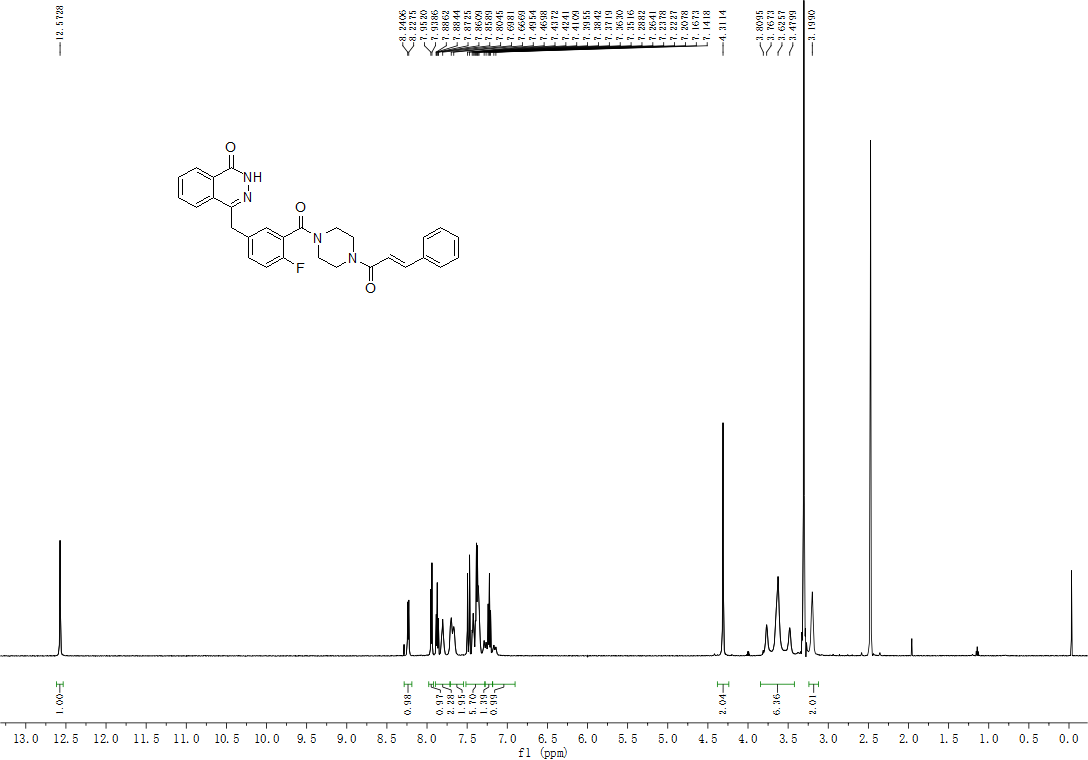


**
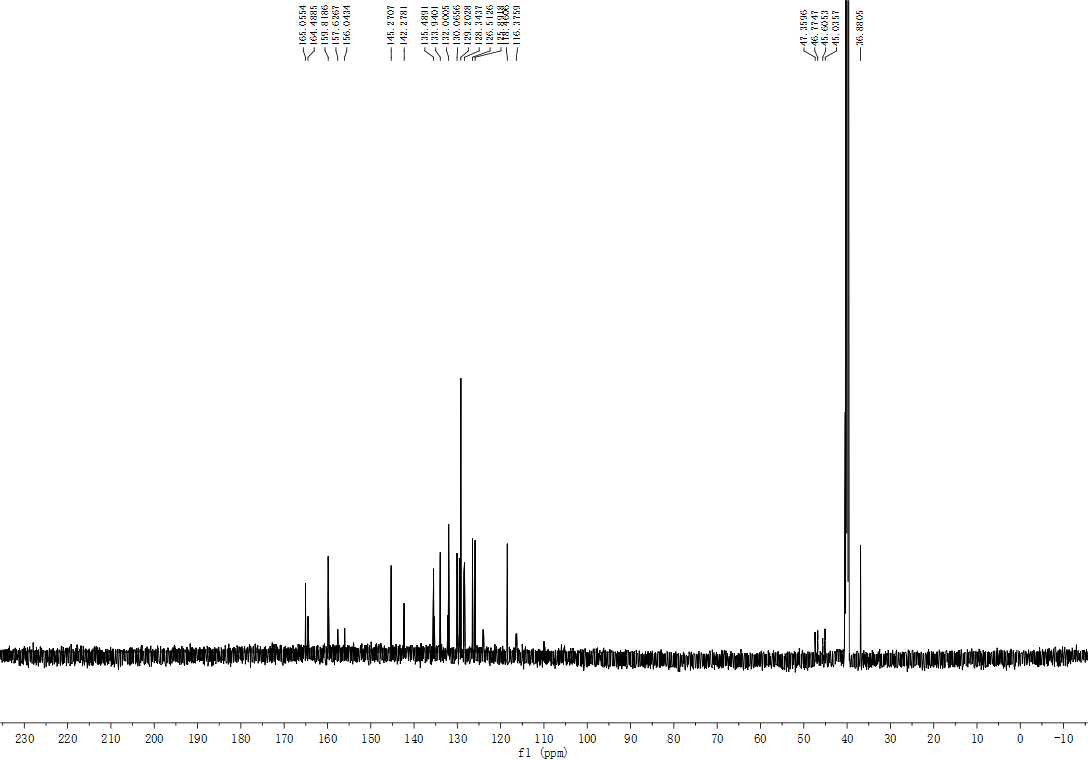
**

***(E)-4-{[3-[4-(3-(3,4-dimethoxyphenyl)acryloyl)piperazine-1-carbonyl]-4-fluorophenyl]methyl}-2H-***

***phthalazin-1-one (5b)***

***
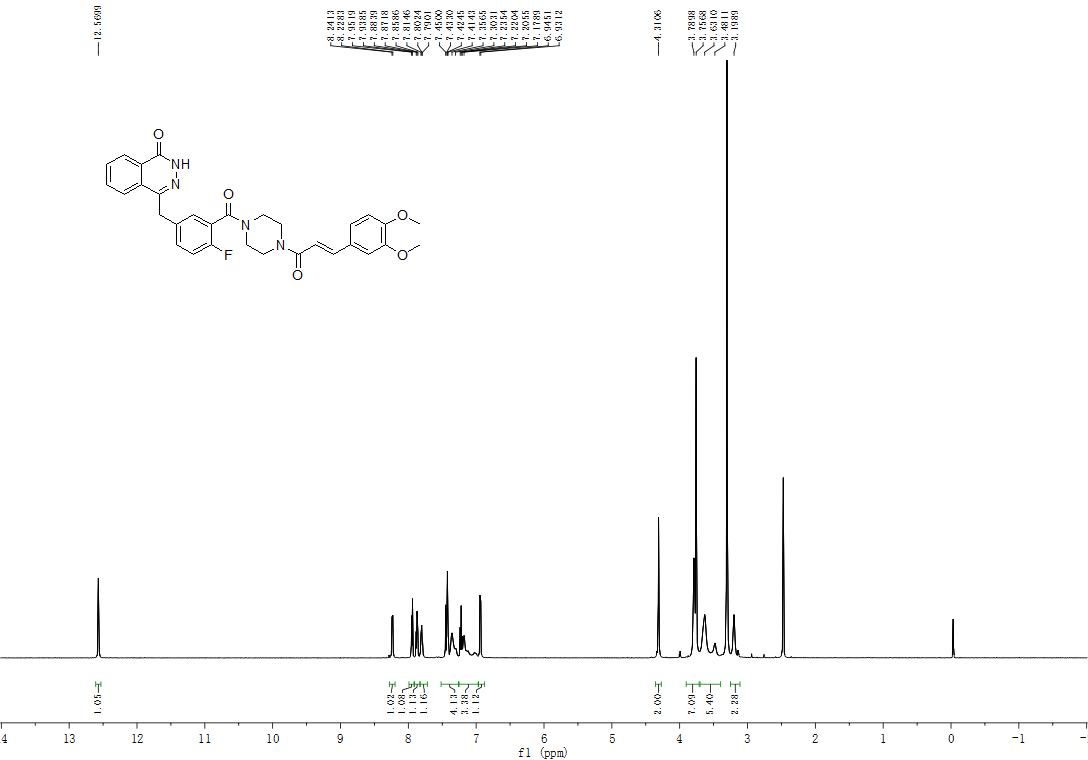
***

**
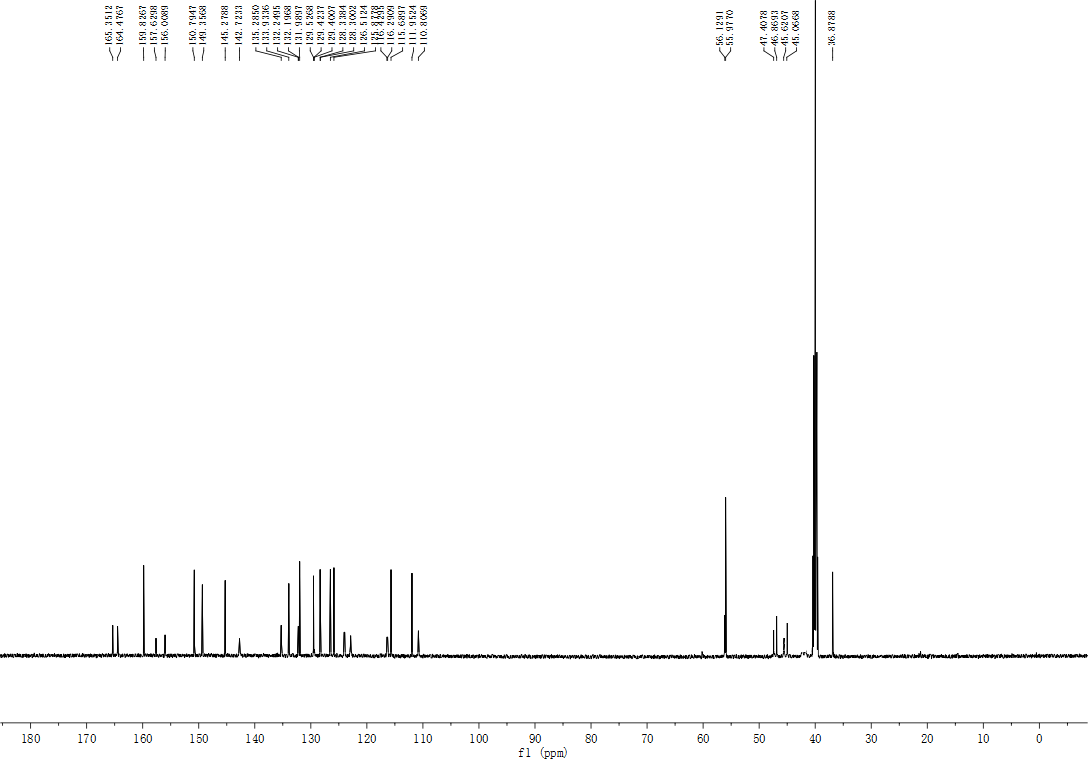
**

***(E)-4-{[3-[4-(3-(4-methoxyphenyl) acryloyl) piperazine-1-carbonyl]-4-fluorophenyl] methyl} -2H-phthalazin-1-one (5c)***

**
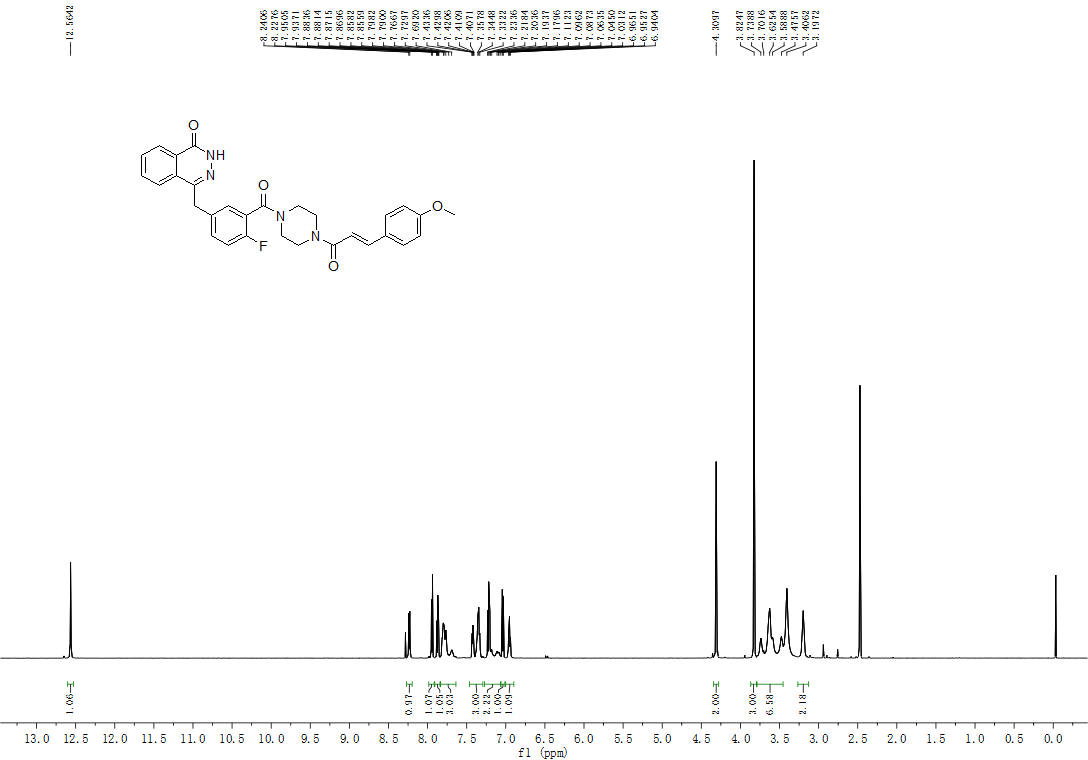
**

**
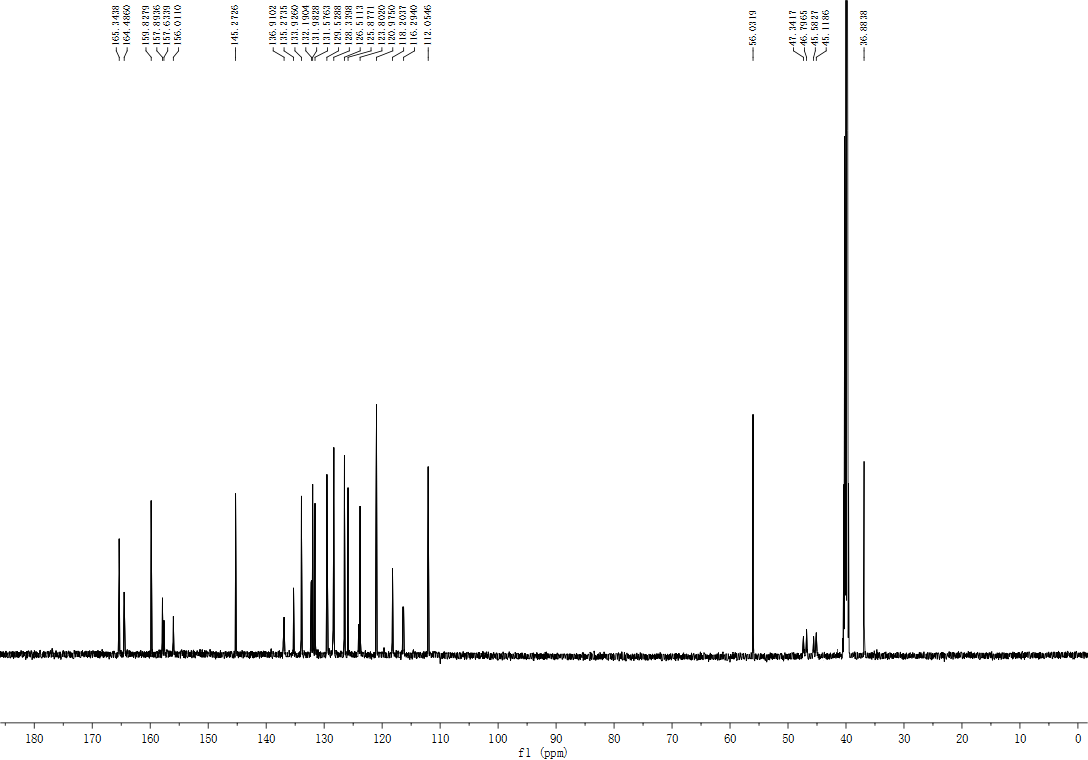
**

***(E)-4-{[3-[4-(3-(4-methylphenyl) acryloyl) piperazine-1-carbonyl]-4-fluorophenyl] methyl} -2H-phthalazin-1-one (5d)***

**
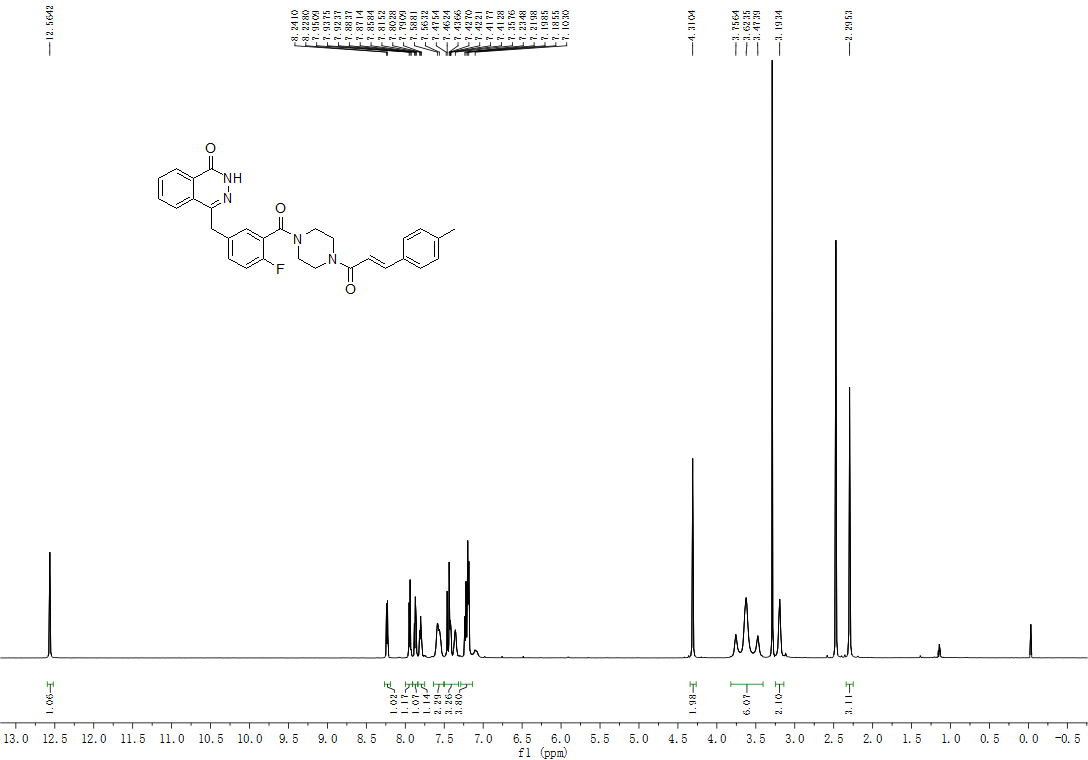
**

**
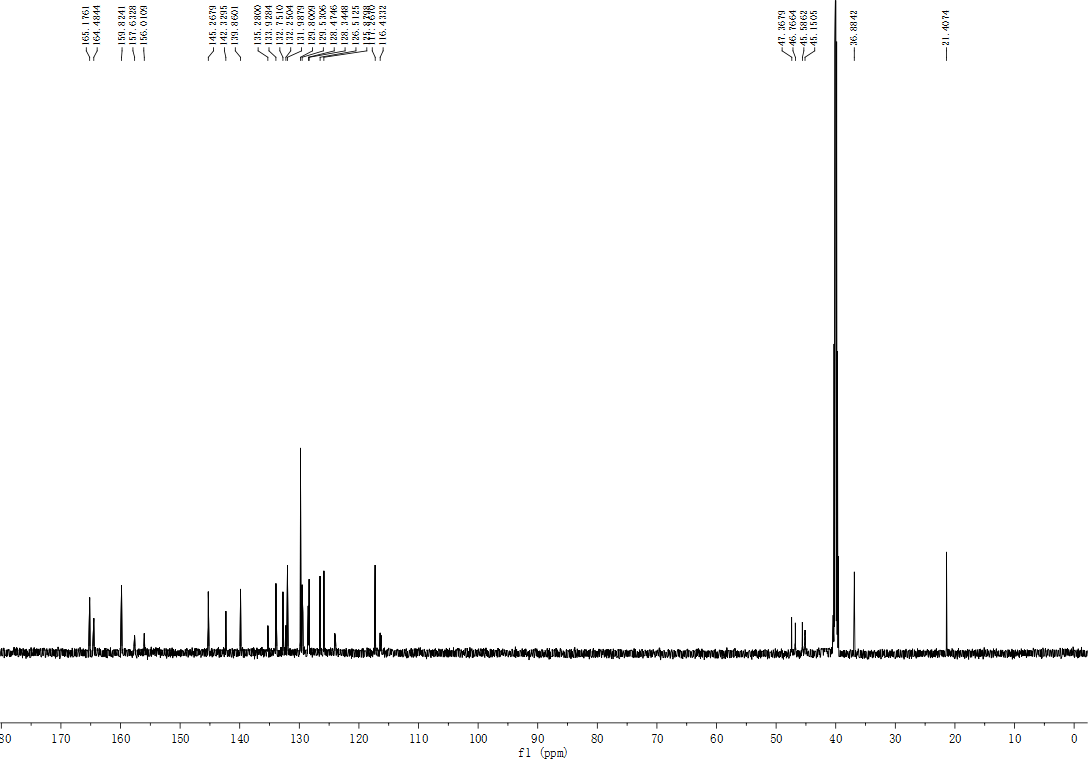
**

***(E)-4-{[3-[4-(3-(2-methoxyphenyl)acryloyl)piperazine-1-carbonyl]-4-fluorophenyl]methyl}-2H-***

***phthalazin-1-one (5e)***

**
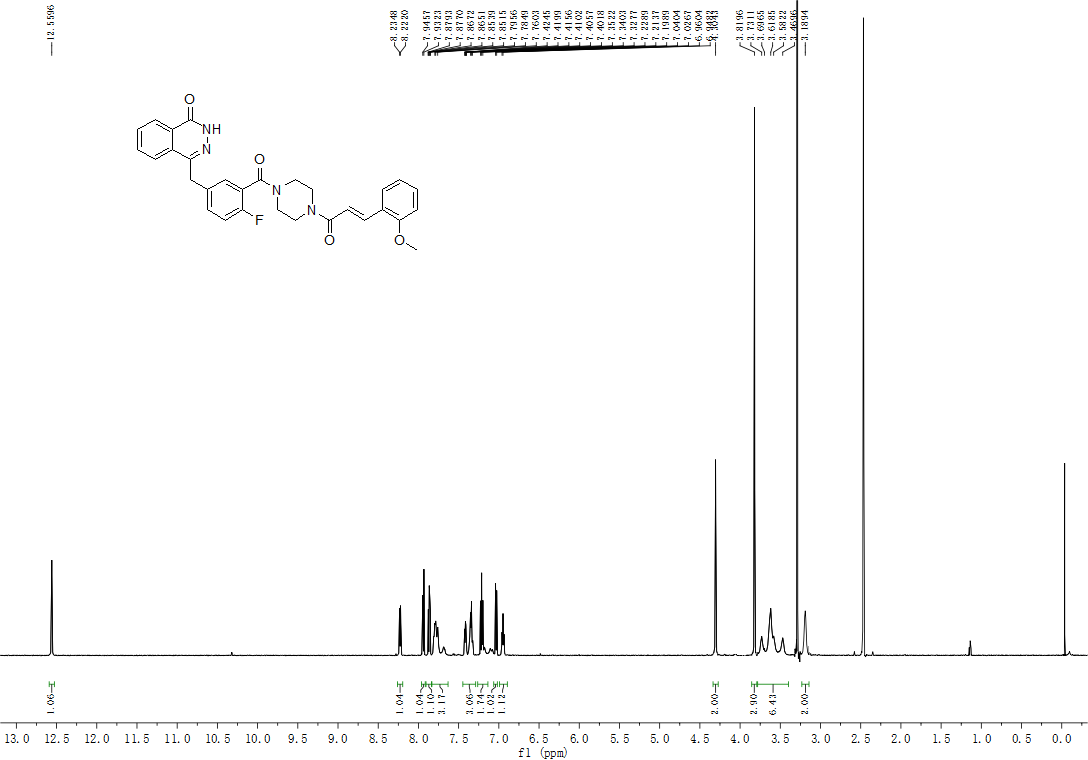
**

**
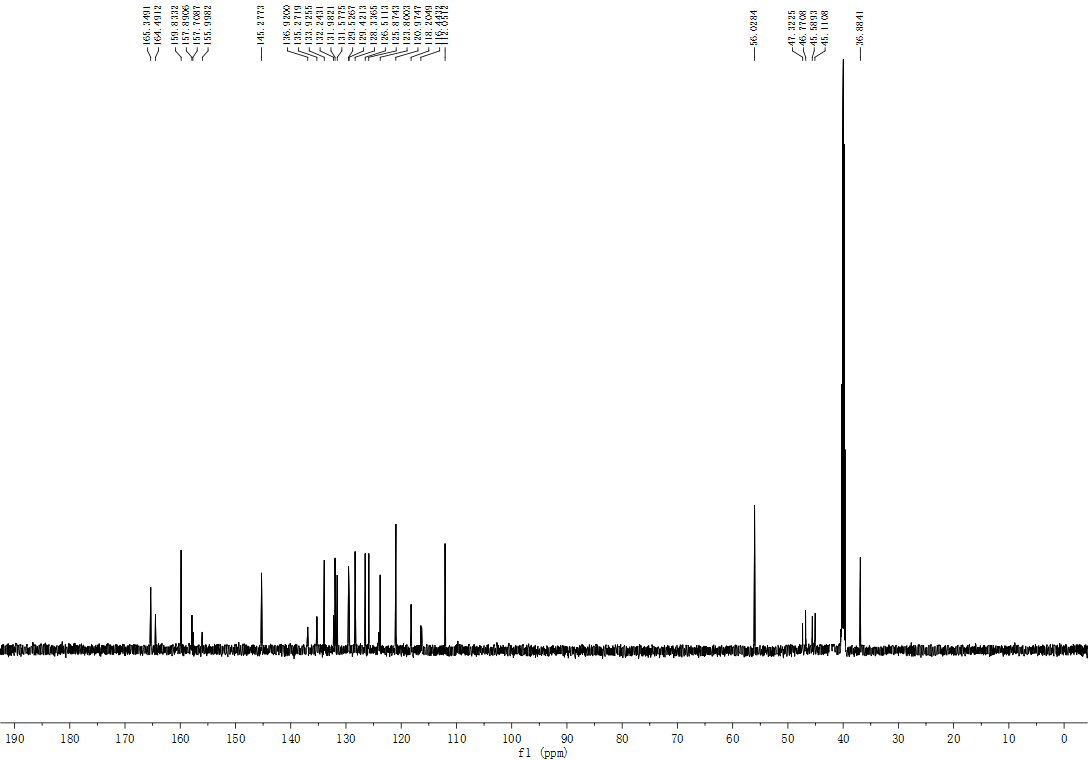
**

***(E)-4-{[3-[4-(3-(2, 5-dimethoxyphenyl) acryloyl) piperazine-1-carbonyl]-4-fluorophenyl] methyl} -2H-phthalazin-1-one (5f)***

**
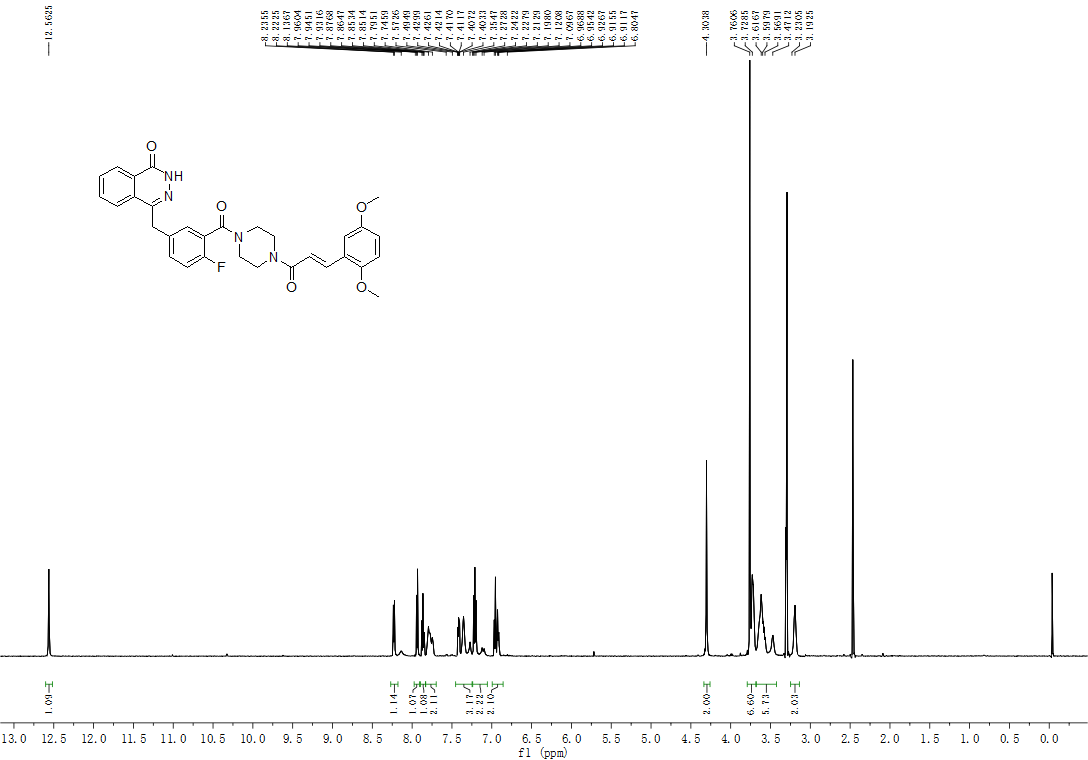
**

**
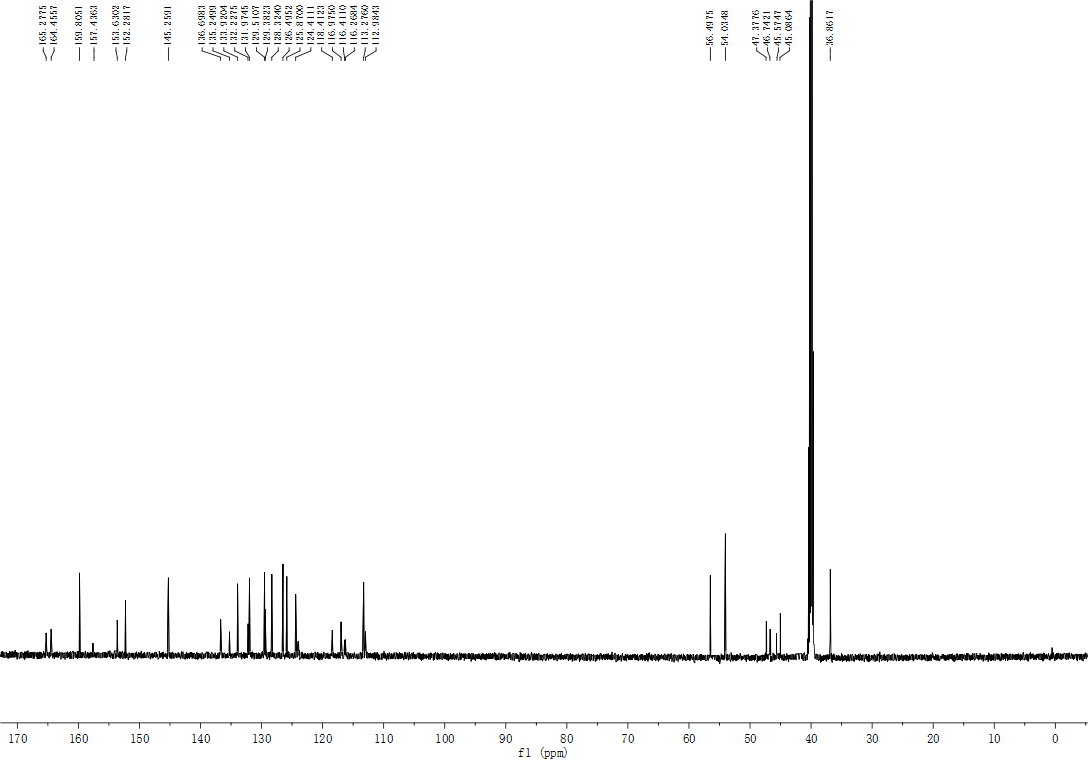
**

***(E)-4-{[3-[4-(3-(4-fluorophenyl) acryloyl) piperazine-1-carbonyl]-4-fluorophenyl] methyl} -2H -phthalazin-1-one (5g)***

***
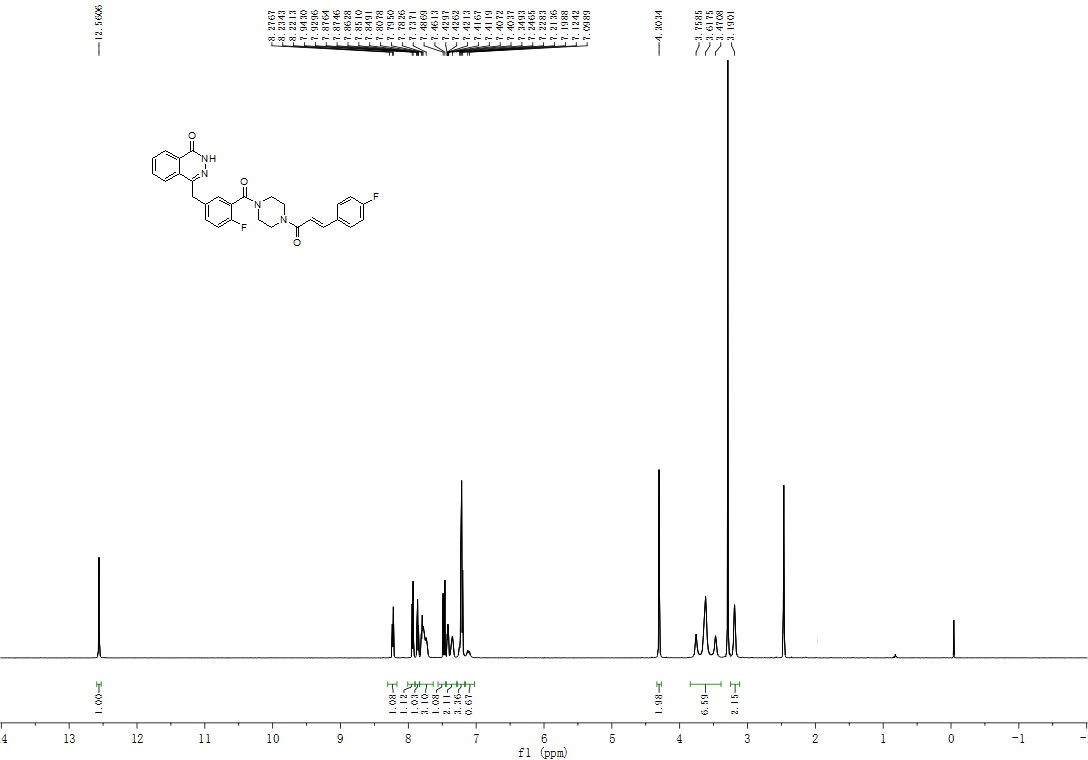
***

***
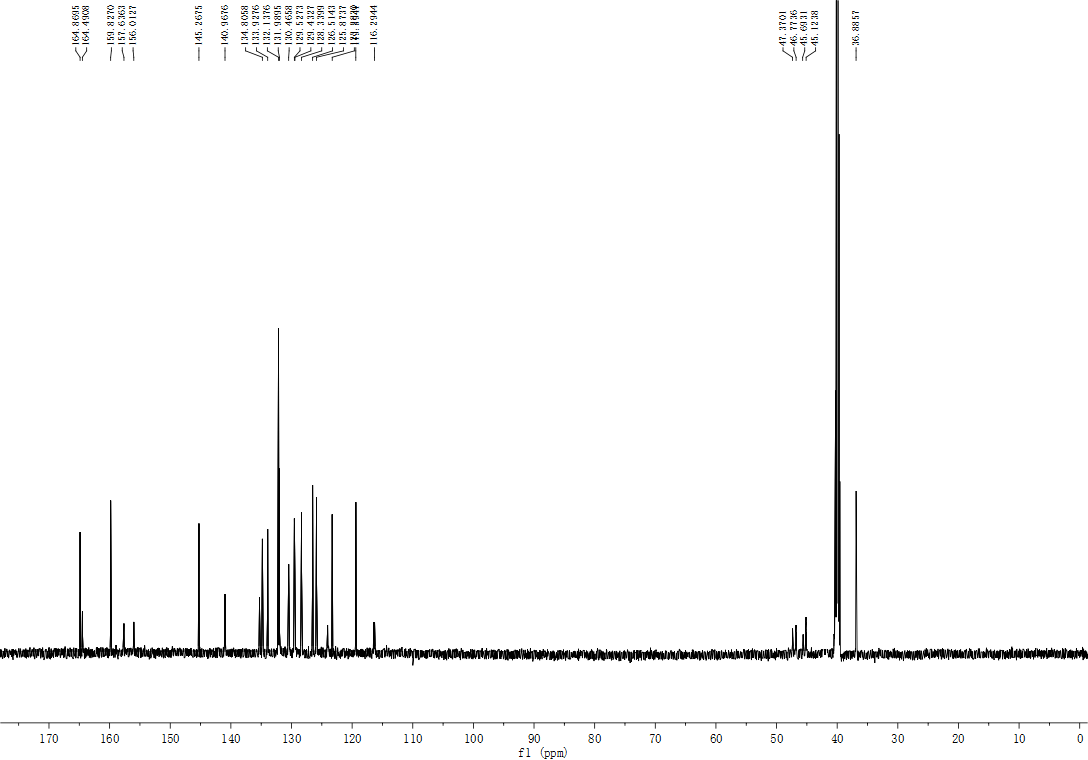
***

***(E)-4-{[3-[4-(3-(4-trifluoromethylphenyl) acryloyl) piperazine-1-carbonyl]-4-fluorophenyl] methyl} -2H -phthalazin-1-one (5h)***

**
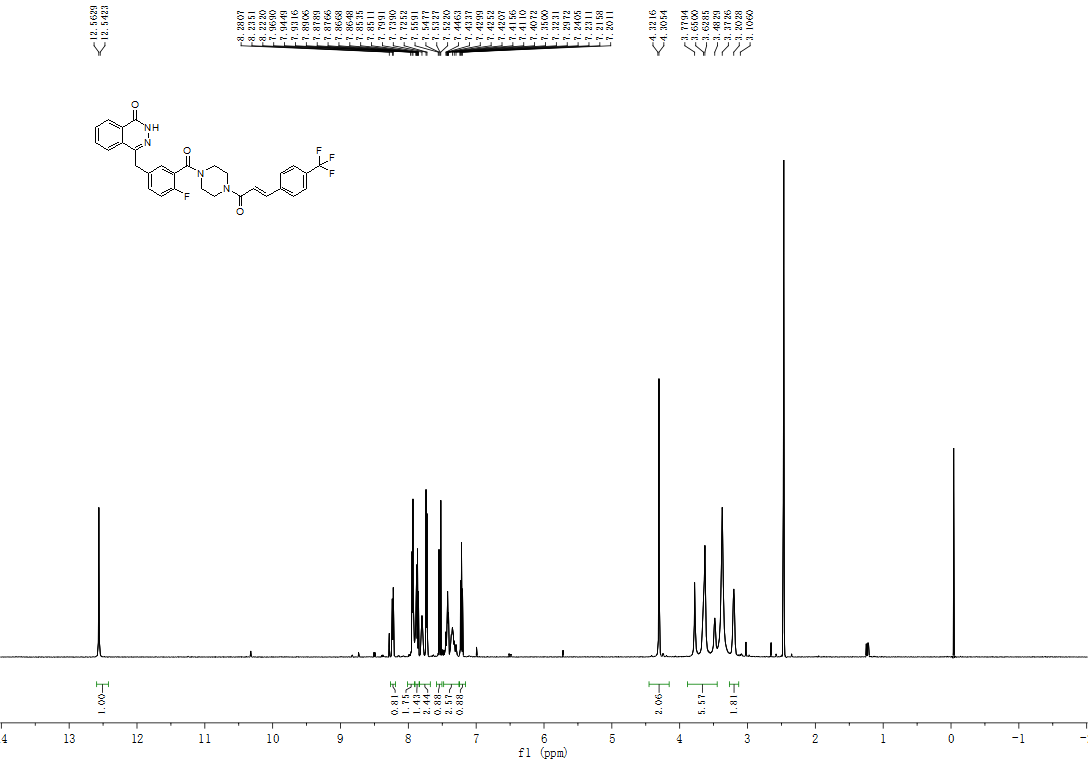
**

**
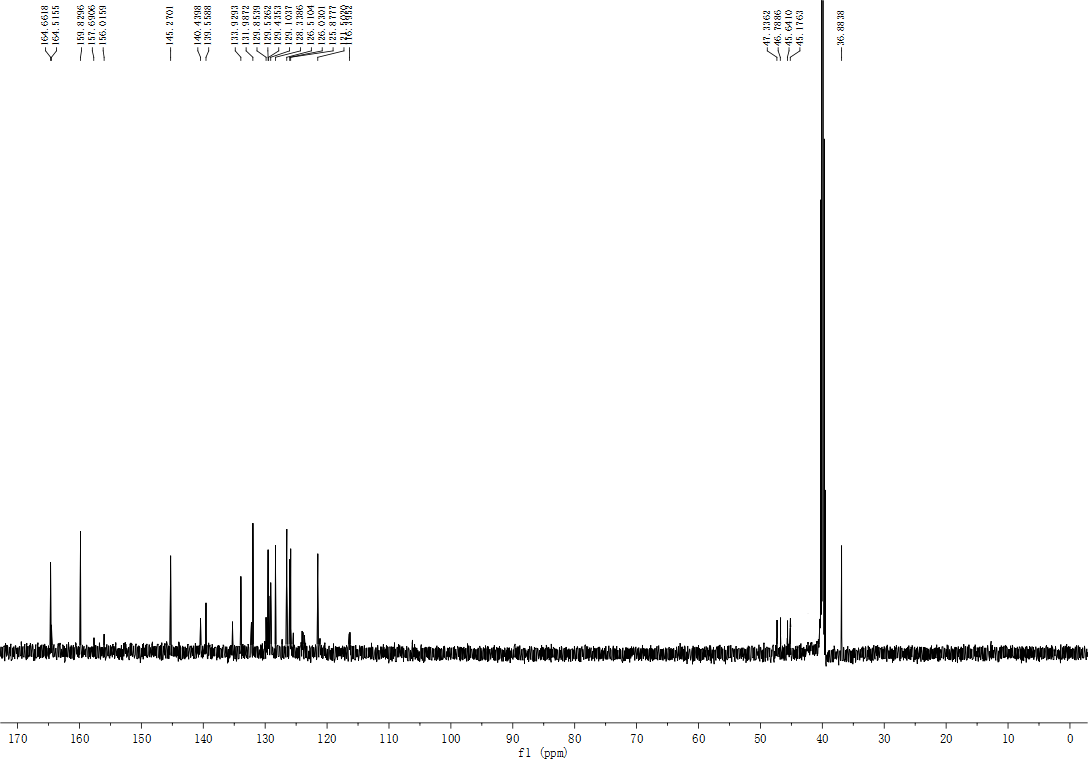
**

***(E)-4-{[3-[4-(3-(3-fluorophenyl) acryloyl) piperazine-1-carbonyl]-4-fluorophenyl] methyl} -2H -******phthalazin-1-one (5i)***

**
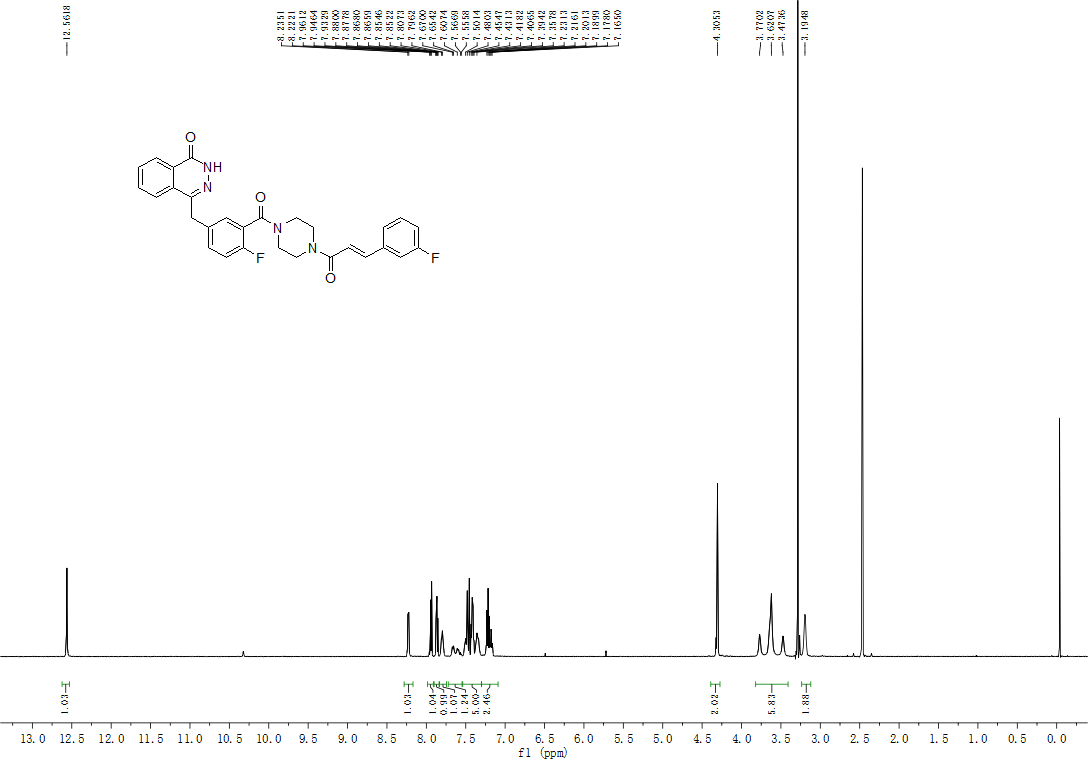
**

**
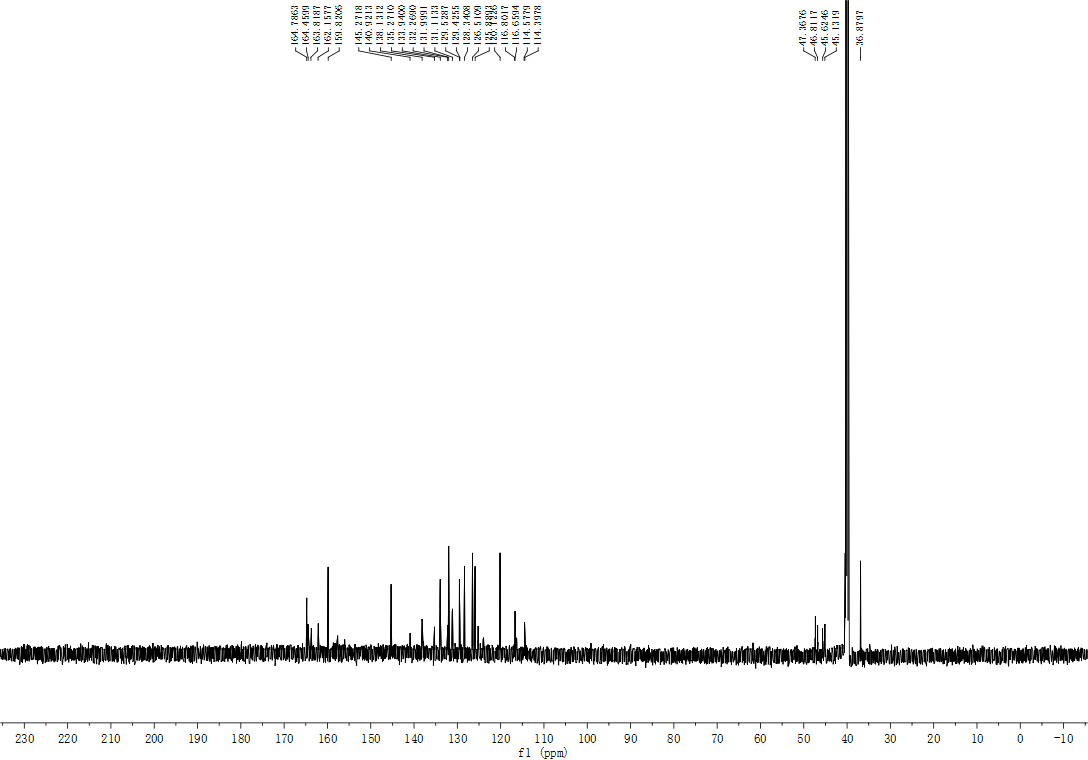
**

***(E)-4-{[3-[4-(3-(4-chlorophenyl) acryloyl) piperazine-1-carbonyl]-4-fluorophenyl] methyl} -2H -phthalazin-1-one (5j)***

**
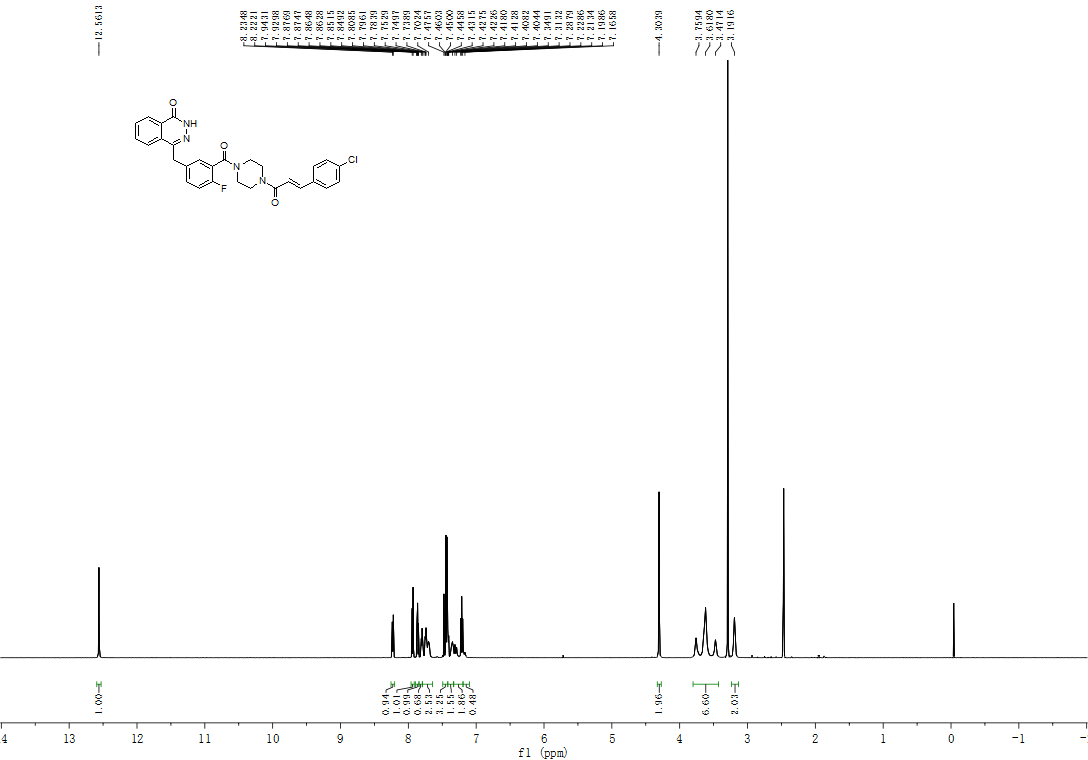
**

**
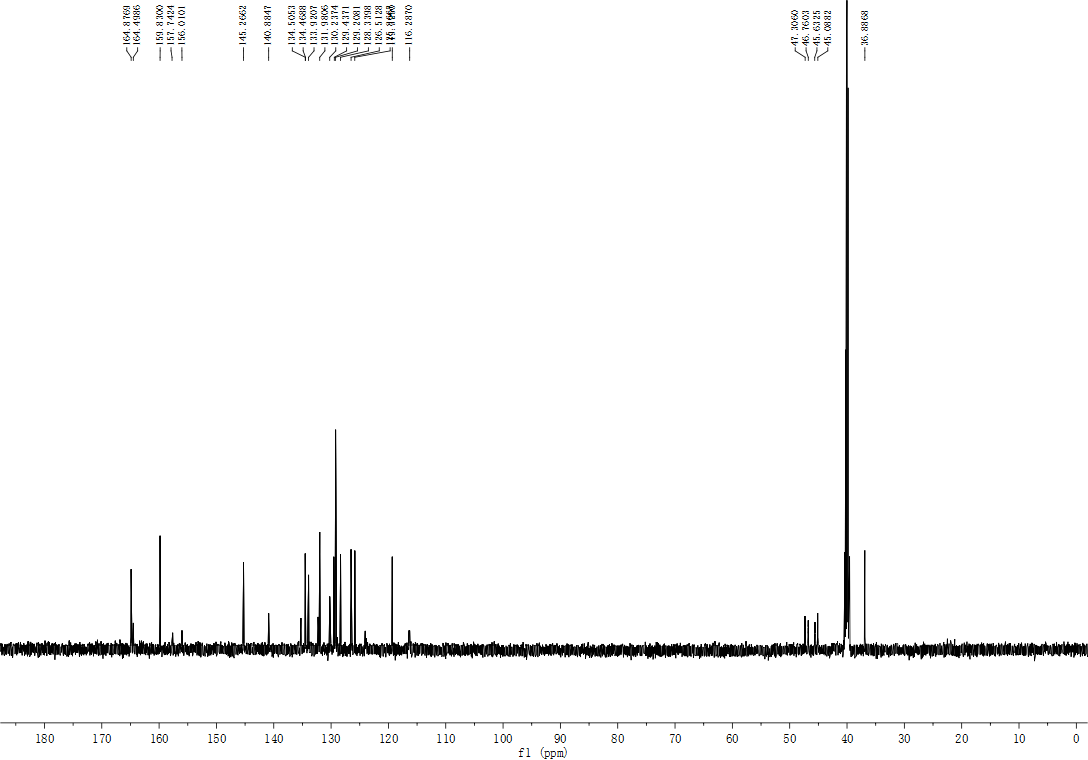
**

***(E)-4-{[3-[4-(3-(4-bromophenyl) acryloyl) piperazine-1-carbonyl]-4-fluorophenyl] methyl} -2H -phthalazin-1-one (5k)***

**
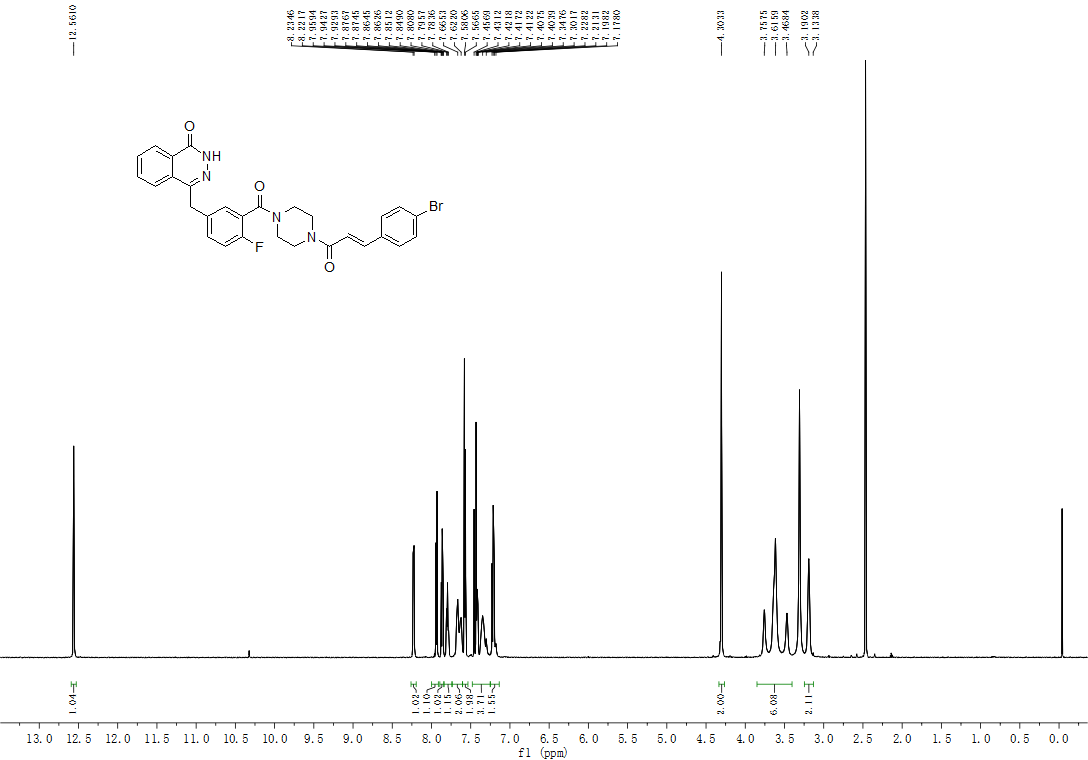
**

**
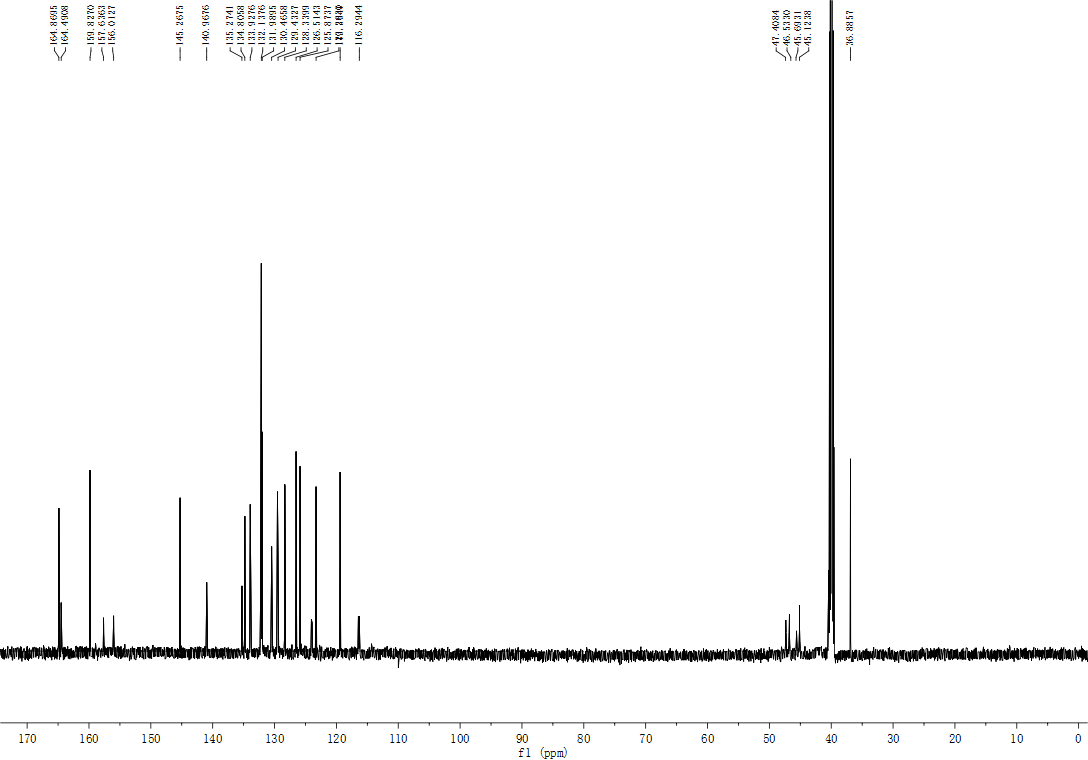
**

***(E)-4-{[3-[4-(3-(3-nitrophenyl) acryloyl) piperazine-1-carbonyl]-4-fluorophenyl] methyl} -2H -phthalazin-1-one (5l)***

**
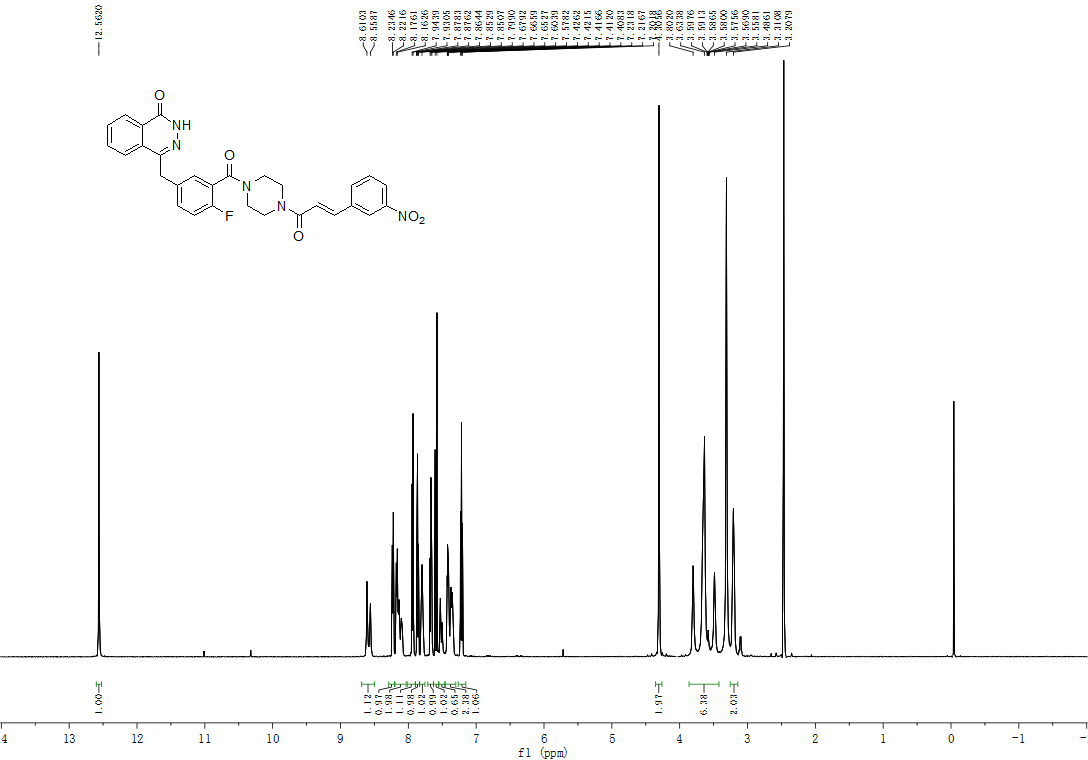
**

**
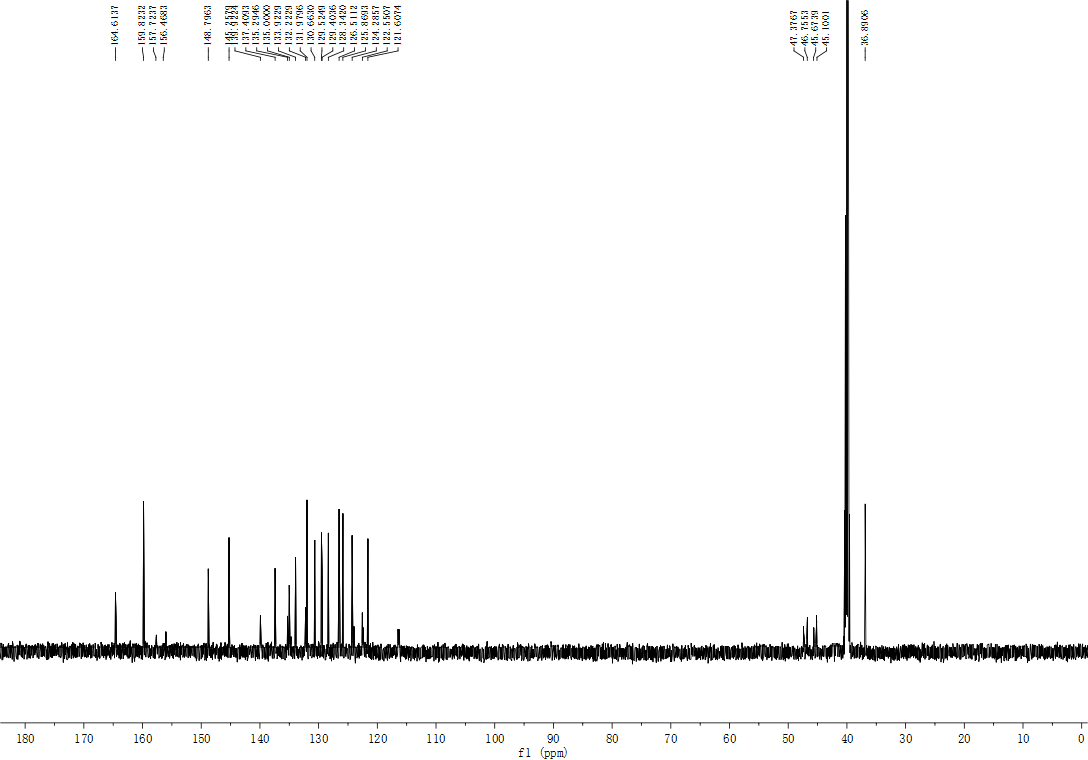
**

***(E)-4-{[3-[4-(3-(4-nitrophenyl) acryloyl) piperazine-1-carbonyl]-4-fluorophenyl] methyl} -2H -phthalazin-1-one (5m)***

**
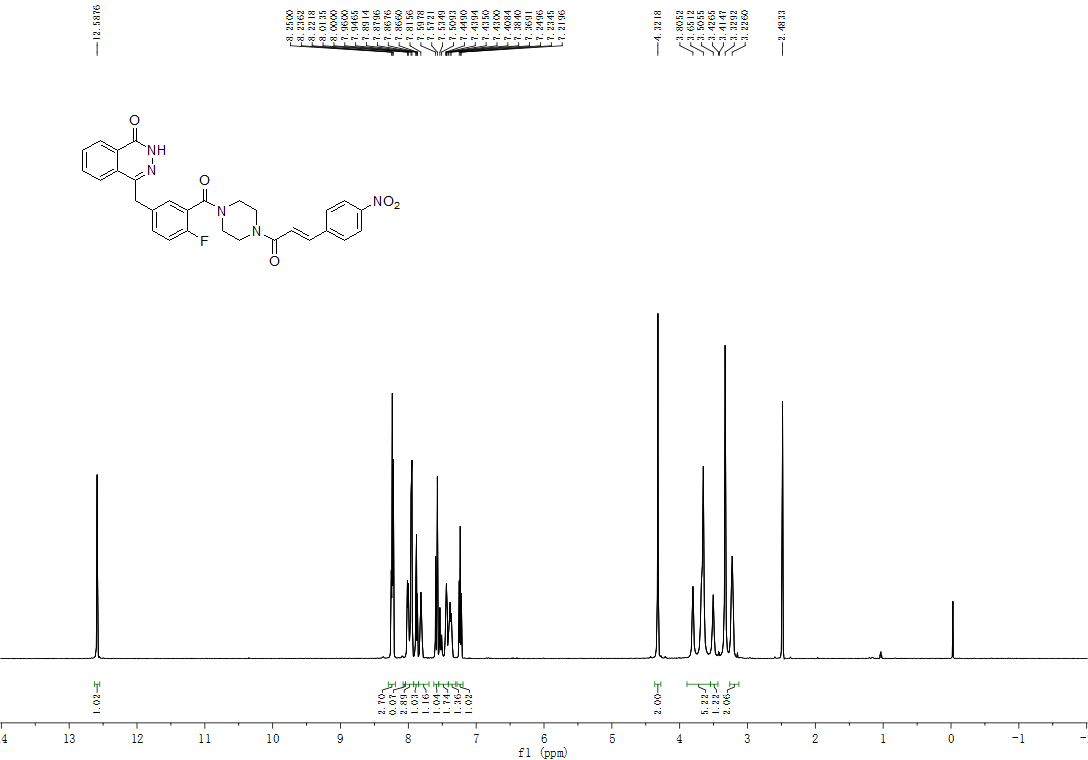
**

**
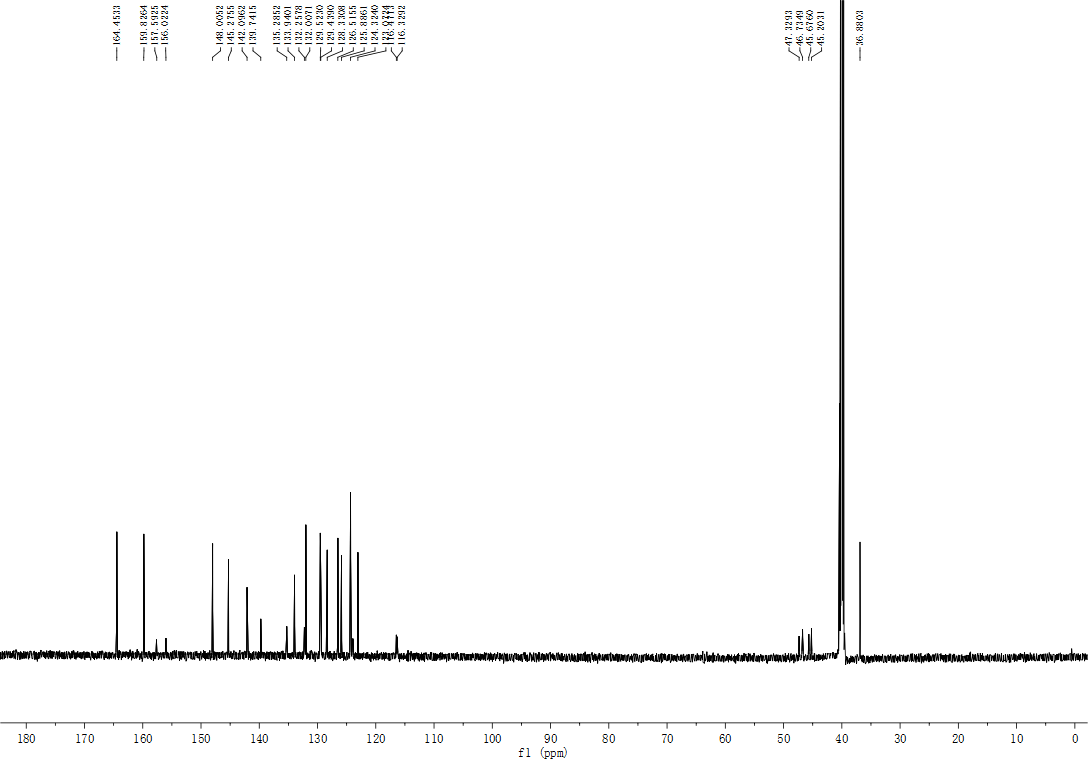
**

***(E)-4-{[3-[4-(3-(3, 4, 5-trimethoxyphenyl) acryloyl) piperazine-1-carbonyl]-4-fluorophenyl] methyl} -2H -phthalazin-1-one (5n)***

***
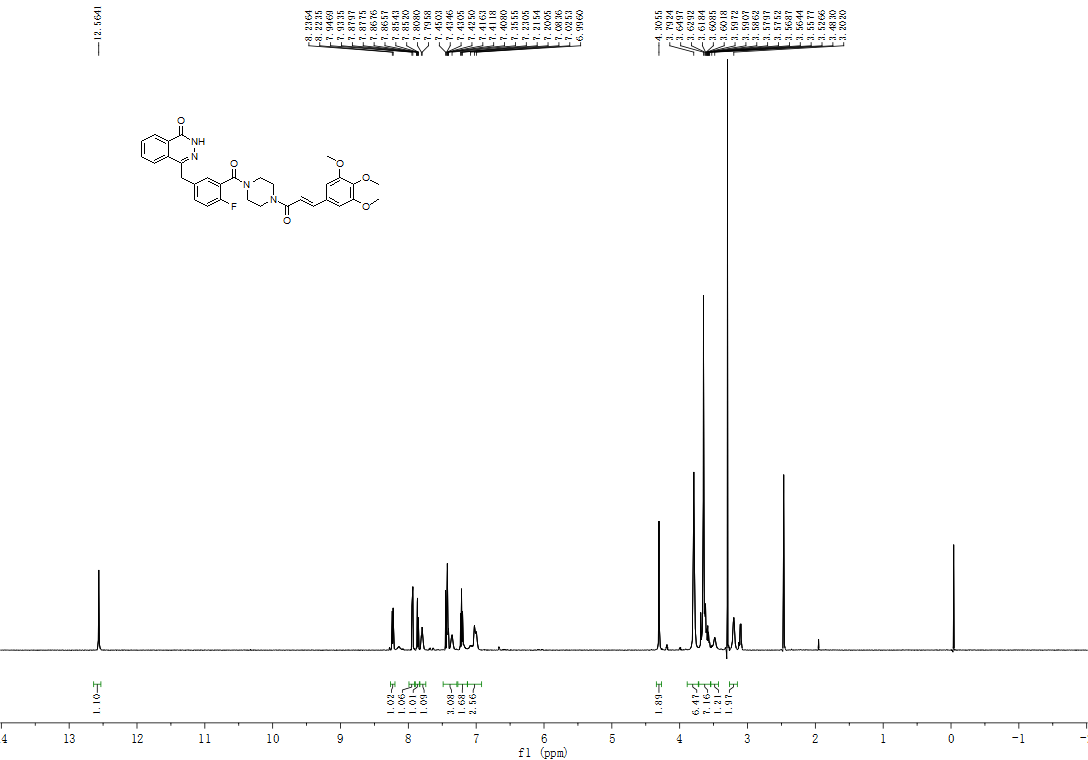
***

***
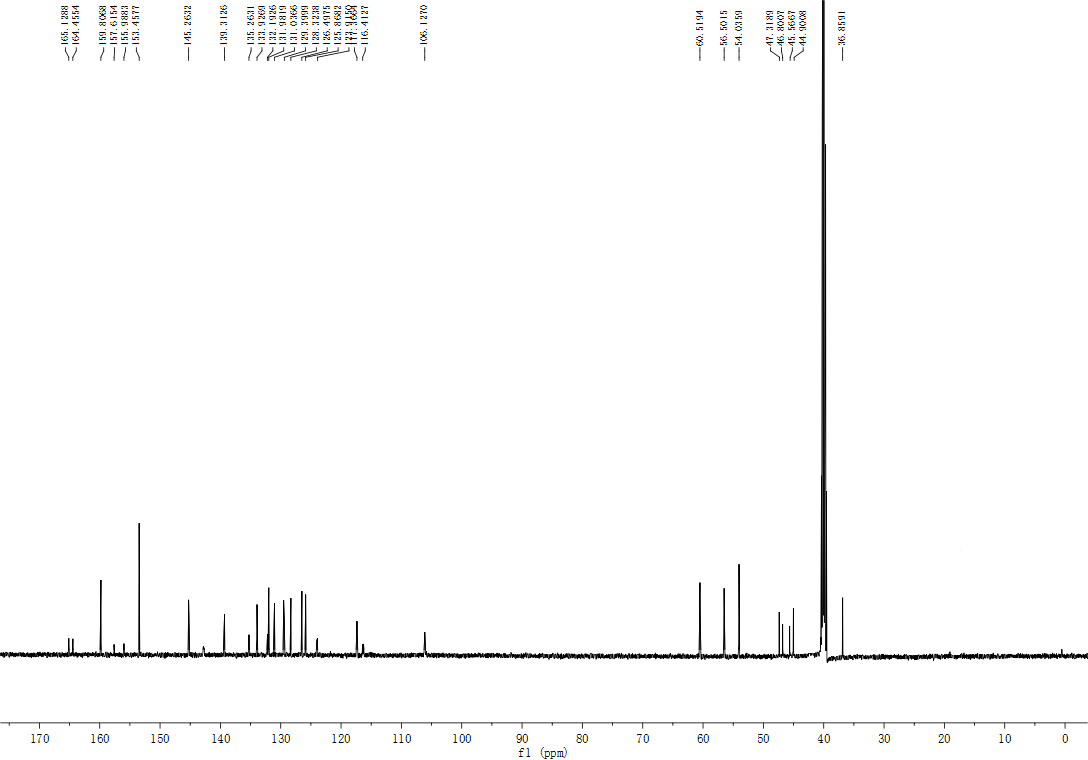
***

***(E)-4-{[3-[4-(3-(furan-2-yl)*** ***acryloyl) piperazine-1-carbonyl]-4-fluorophenyl] methyl} -2H -phthalazin-1-one (5o)***

**
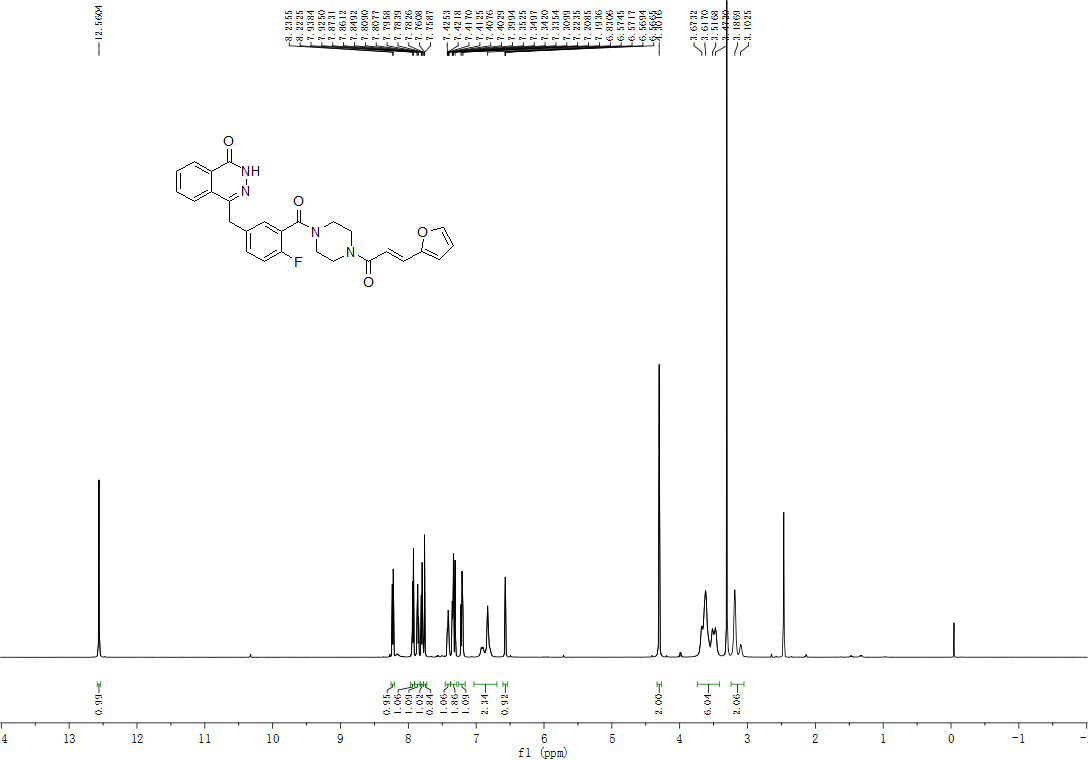
**

**
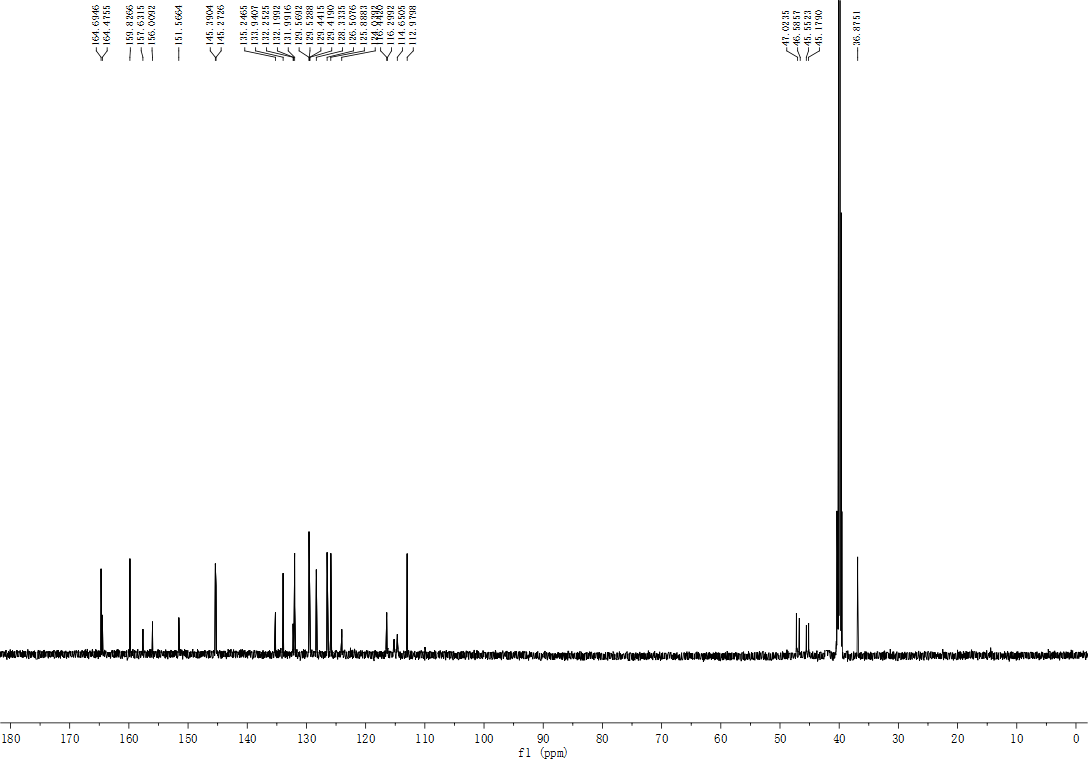
**
